# Spectral and DFT studies of anion bound organic receptors: Time dependent studies and logic gate applications

**DOI:** 10.3762/bjoc.13.25

**Published:** 2017-02-06

**Authors:** Srikala Pangannaya, Neethu Padinchare Purayil, Shweta Dabhi, Venu Mankad, Prafulla K Jha, Satyam Shinde, Darshak R Trivedi

**Affiliations:** 1 Supramolecular Chemistry Laboratory, Department of Chemistry, National Institute of Technology Karnataka (NITK), Surathkal, India; 2 Department of Physics, Maharaja Krishnakumarsinhji Bhavnagar University, Bhavnagar 364001, India; 3 Department of Physics, Faculty of Science, The M.S. University of Baroda, Vadodara 390002, India; 4 School of Technology, Pandit Deendayal Petroleum University, Gandhinagar 382007, Gujarat, India

**Keywords:** colorimetric sensor, DFT, molecular logic gates, rate constant, substituent effect

## Abstract

New colorimetric receptors **R1** and **R2** with varied positional substitution of a cyano and nitro signaling unit having a hydroxy functionality as the hydrogen bond donor site have been designed, synthesized and characterized by FTIR, ^1^H NMR spectroscopy and mass spectrometry. The receptors **R1** and **R2** exhibit prominent visual response for F^−^ and AcO^–^ ions allowing the real time analysis of these ions in aqueous media. The formation of the receptor–anion complexes has been supported by UV–vis titration studies and confirmed through binding constant calculations. The anion binding process follows a first order rate equation and the calculated rate constants reveal a higher order of reactivity for AcO^−^ ions. The ^1^H NMR titration and TDDFT studies provide full support of the binding mechanism. The Hg^2+^ and F^−^ ion sensing property of receptor **R1** has been utilized to arrive at “AND” and “INHIBIT” molecular logic gate applications.

## Introduction

The development of new organic receptors for the detection of anions is of key interest to supramolecular chemists owing to the biological and environmental importance of anions [1–7]. The leading role of anions such as fluoride, acetate and phosphate at the physiological level in promoting tooth and bone health, metabolism and genetic transduction has been well established [8–14]. Increasing research interest on the selective and sensitive detection of anions has enriched the field of anion receptor chemistry with a wide array of design strategies [15–19]. Among the various analytical techniques, colorimetry has drawn significant attention among chemists for its rapid response rate, low cost, easy method and high selectivity [20–26]. The choice of the appropriate detection technique is highly essential as it directly dictates the efficacy of the sensor.

Anion binding through colorimetric probes comprising of a binding site and a signaling unit works in a coordinative way yielding an optical output visible to the naked eye. The detection of anions is commonly encountered with challenges in the receptor–anion interactions such as size and shape effects, pH, and solvation effects. In this regard, considerable efforts have been devoted towards the design of suitable receptors in the past few decades. Numerous receptors for anions have been developed based on various modes of interactions such as hydrogen bond and electrostatic interactions which rely on directionality and distance-dependent nature, respectively. Hydrogen bond formation is further tuned by the acidity of protons by virtue of the presence of electron-withdrawing substituents [1]. Pyridine-based derivatives have been designed by researchers in the context of detection of anions involving hydrogen bonding and a deprotonation mechanism. Gunnlaugsson and co-workers have reported a pyridine-based thiosemicarbazide derivative for the detection of OH^−^, F^−^ and AcO^−^ ions through hydrogen bond interaction followed by a deprotonation process [27–29].

The design of molecular logic gates with chemical and biological molecules has been at the forefront creating a new avenue to advanced diagnostics and therapeutics through molecular computers. As an added advantage, in molecularly gated devices, Boolean logic computations could be activated by specific inputs and accurately processed through biorecognition, biocatalysis and selective chemical reactions [30]. The utilization of designed receptors in molecular logic gate applications has seen great progress ever since the first AND logic gate was mimicked with optical signals by de Silva and co-workers [31]. Myriads of chemical systems have been used by researchers towards the development of different functions such as AND, OR, NOT and their integrated operations [32]. Moreover the receptors with a multiple input molecular logic gate are gaining more interest as they are known to perform special arithmetic operations [33–35]. In addition, researchers have implemented integrated logic gates such as INHIBIT, half subtractor, half adder, full adder, and full subtractor with various single molecules [36–37].

In this direction, we report the design and synthesis of two new organic receptors decorated with suitable electron-withdrawing substituents viz. a cyano and nitro functionality as signaling unit on the heterocyclic ring. With a vision towards the enhancement of the chromogenic signaling output, the signaling unit has been linked to a conjugated system possessing a hydroxy functionality which acts as binding site for anions. UV–vis, ^1^H NMR titration studies along with DFT studies of the receptors **R1** and **R2** would help to arrive at the binding mechanism. The presence of heteroatoms in the receptors could further allow their use for detecting cations. This dual ion sensing property is expected to play a role in the study of logical interpretations at the arithmetic level.

## Results and Discussion

### UV–vis spectrophotometric studies

Receptors **R1**and **R2** vary in the substituent groups attached to the aromatic ring and possess hydrogen-bond donor functionality, namely a hydroxy group in the naphthyl part, which can act as an active binding site for anions. Additionally, both receptors **R1** and **R2** encompass an electron-withdrawing substituent, a CN group (**R1**) or a NO_2_ functionality (**R2**), in the para position of the imine linkage connecting the conjugated naphthyl group. The pyridine ring possessing –CN and –NO_2_ functionalities in **R1** and **R2** reflect their identity as a signaling unit/chromophore. In total, the nature and position of binding site and signaling unit play the key role in the chromogenic response of the anion detection process.

The anion binding properties of receptors **R1** and **R2** (4.5 × 10^−5^ M in DMSO), have been studied through the addition of 2 equiv of a series of anions as their tetrabutylammonium salts (F^−^, Cl^−^, Br^−^, I^−^, NO_3_^−^, HSO_4_^−^, H_2_PO_4_^−^ and AcO^−^ at concentrations of 1 × 10^−2^ M in DMSO).

**Figure 1:**
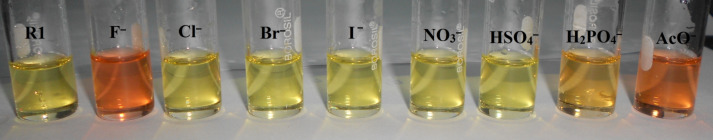
Color change observed for **R1** (4.5 × 10^−5^ Min DMSO) in the presence of 1 equiv of different anions (1 × 10^−2^ M in DMSO).

**Figure 2:**
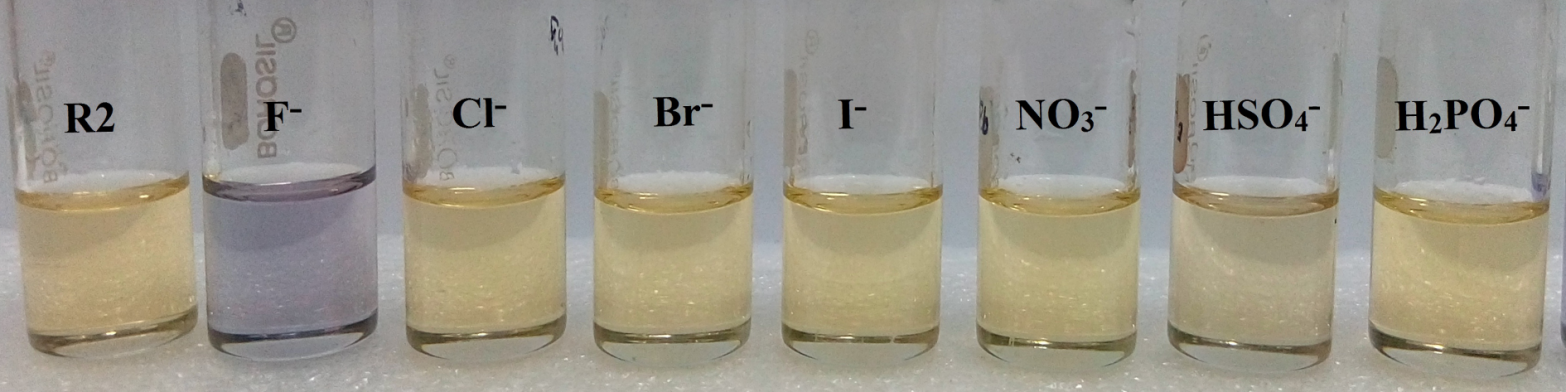
Color change observed for **R2** (4.5 × 10^−5^ M in DMSO) in the presence of 1 equiv of different anions (1 × 10^−2^ M in DMSO).

Both receptors **R1** and **R2** exhibited significant color changes from pale yellow to orange and blue, respectively, in the presence of F^−^ and AcO^−^ ions. The color changes upon the addition of the different anions to solutions of **R1** and **R2** are shown in Figure 1 and Figure 2. The corresponding redshifts in the absorption spectra are shown in Figure S7 and S8 in the Supporting Information File 1.

**Figure 3:**
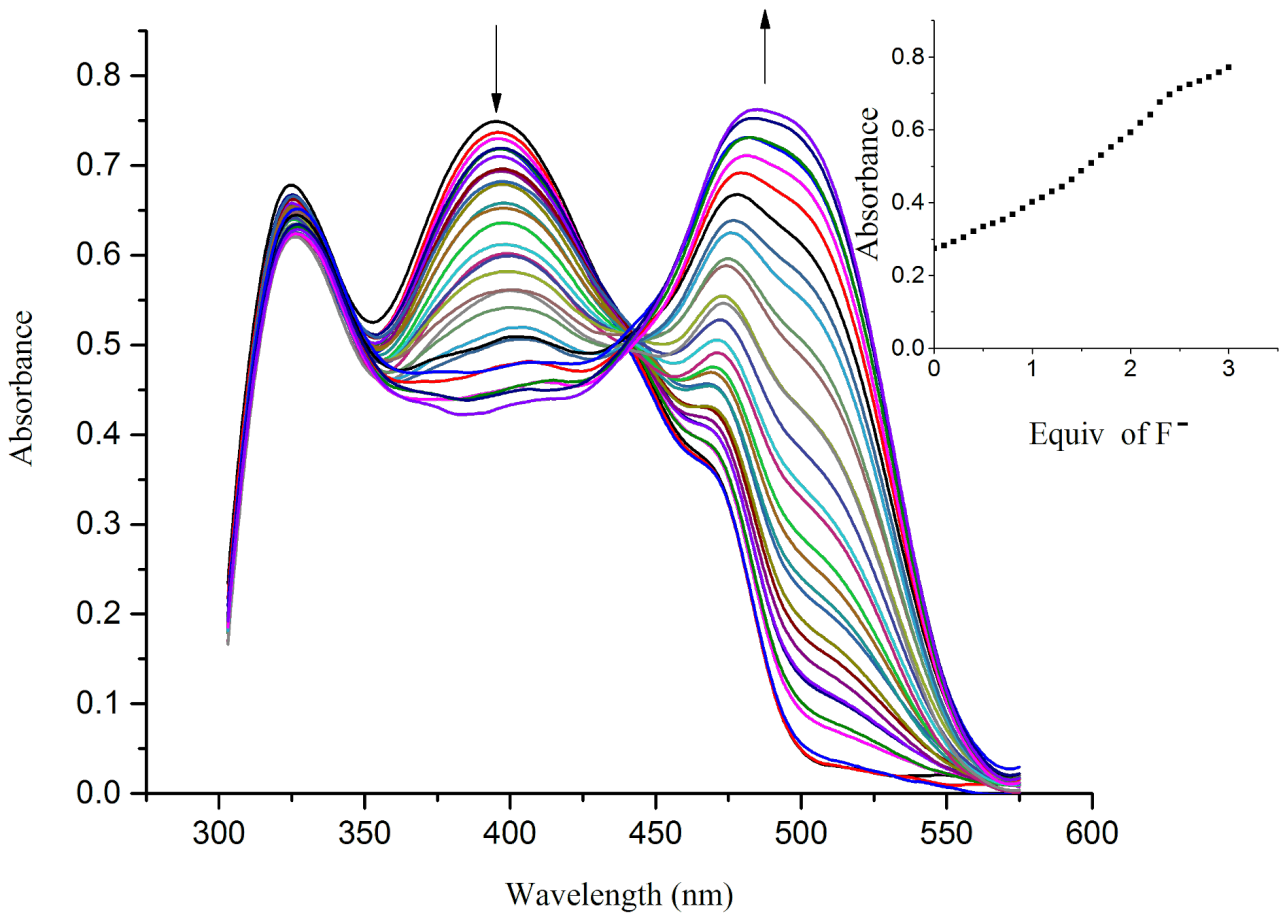
UV–vis titration spectra of receptor **R1** (4.5 × 10^−5^ M in DMSO) obtained by the incremental addition of 0.1 equiv of TBAF (1 × 10^−2^ M in DMSO). The inset plot represents the binding isotherm at 477 nm.

**Figure 4:**
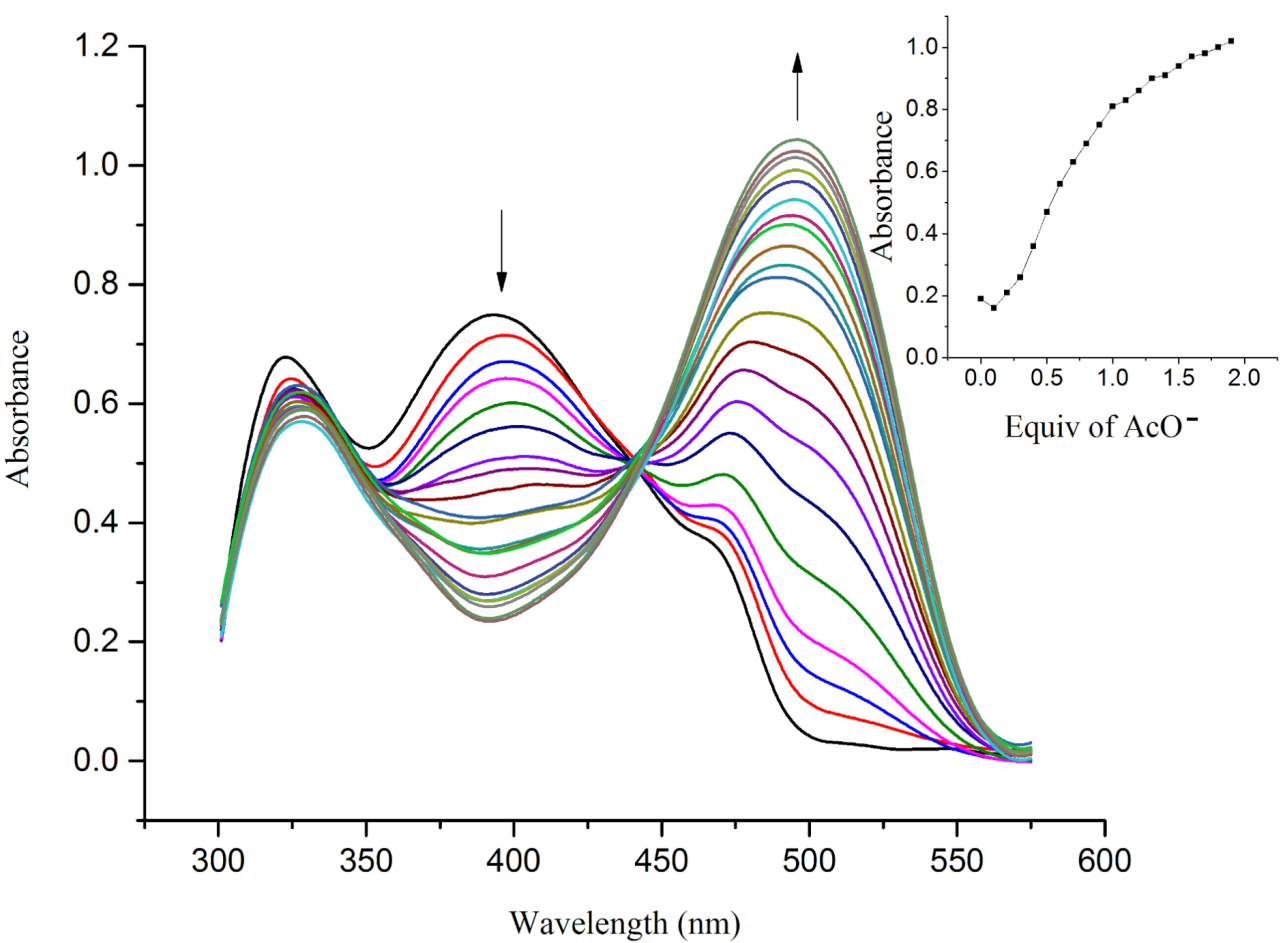
UV–vis titration spectra of receptor **R1** (4.5 × 10^−5^ M in DMSO) obtained by the incremental addition of 0.1 equiv of TBAOAc (1 × 10^−2^ M in DMSO). The inset plot represents the binding isotherm at 492 nm.

UV–vis spectral analyses have been performed to analyze the observed color changes. In the spectrum of the free receptor **R1** (4.5 × 10^−5^ M in DMSO), the absorption bands at 325 nm and 395 nm correspond to the transitions between the π orbital of the azomethine group and the OH functionality involved in an intramolecular charge-transfer process, respectively [38]. The incremental addition of 0.1 equiv of TBA salts of F^−^ and AcO^−^ resulted in a red shift of the original charge-transfer bands to 477 nm and 492 nm, respectively, and are represented in Figure 3 and Figure 4.

**Figure 5:**
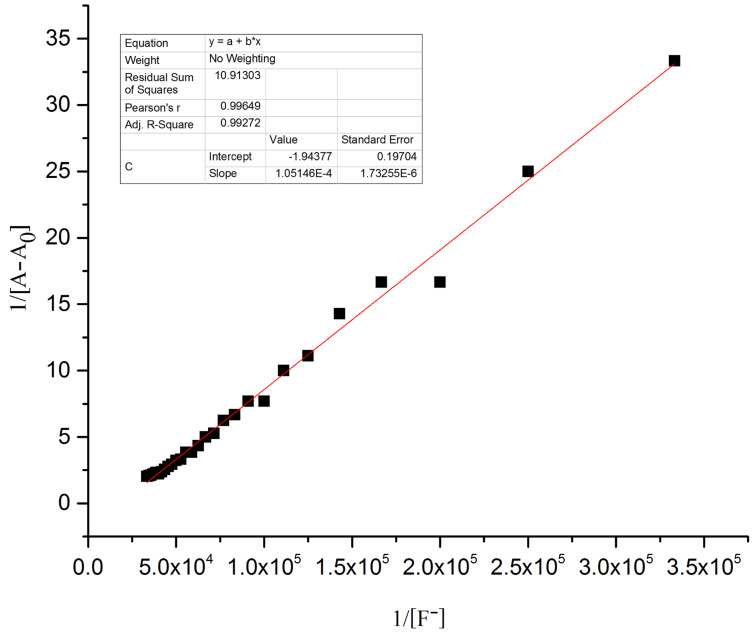
B–H plot of the **R1**–F^−^ complex at a selected wavelength of 477 nm.

**Figure 6:**
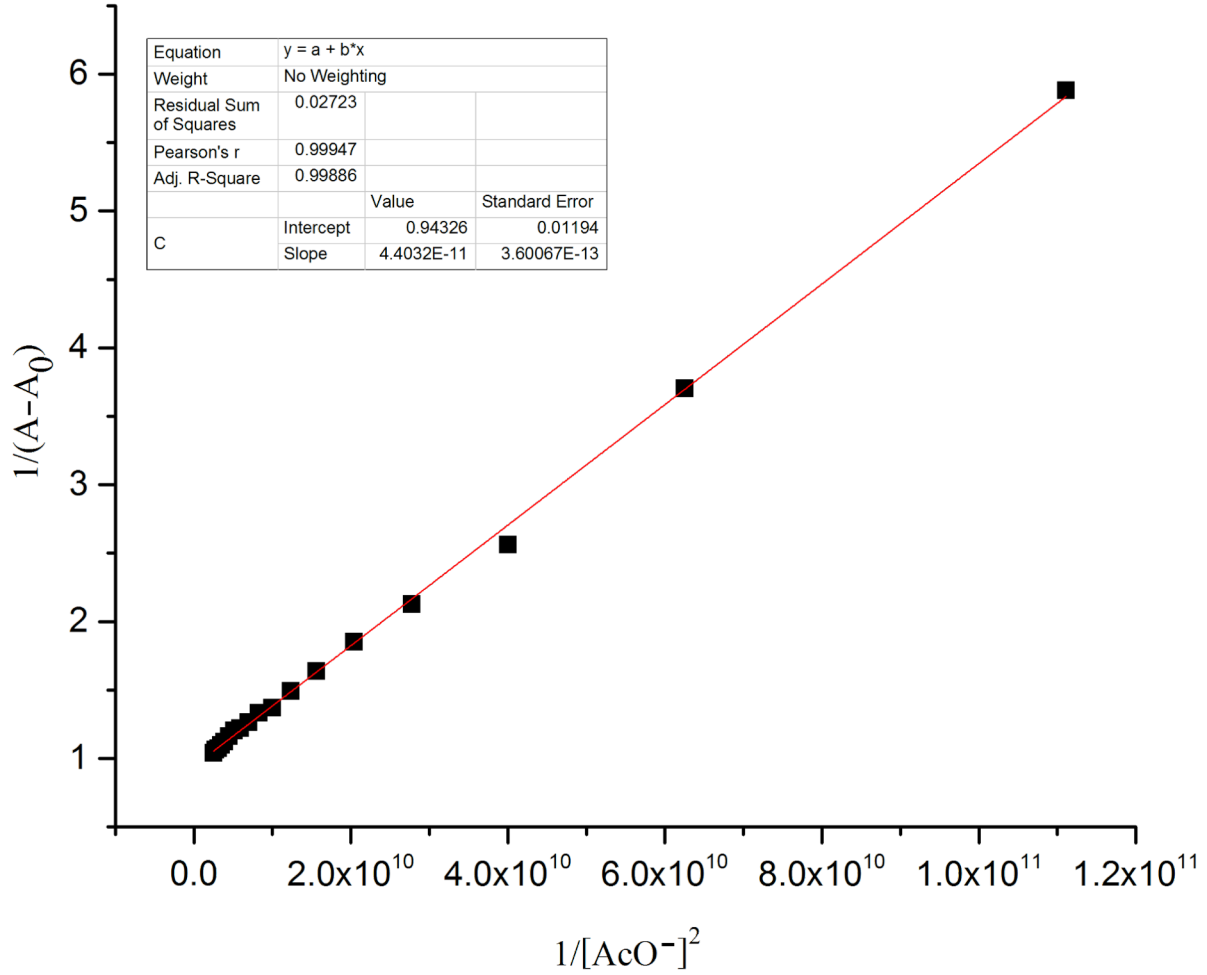
B–H plot of the **R1**–AcO^−^ complex at a selected wavelength of 492 nm.

The hydrogen-bond interaction between the OH group in R1 and the guest anion is assisted through an intermolecular proton transfer (IPT) suggesting a proton abstraction from a OH group and introduction of a negative charge on the oxygen atom. The enhancement of the ICT transition could be ascribed to a push–pull nature between the electron withdrawing –CN substituent and the conjugated system [39]. The appearance of a clear isosbestic point at 443 nm indicates the existence of a host–guest complex in the system. On the other hand, the complete diminution of the peak at 395 nm during the successive addition of AcO^−^ and F^−^ ions clearly supports the deprotonation process and the requirement of 2 equiv of acetate and fluoride ions by receptor **R1** to attain saturation confirms the assumed deprotonation of the OH functionality. Next, the binding ratio was determined by B–H plot. Plotting 1/[A – A_0_] versus 1/[F^−^] resulted in a straight line with first power of concentration of F^−^ ion confirming the binding of receptor **R1** with F^−^ ion in a 1:1 ratio as shown in Figure 5. For acetate binding a linear plot was obtained for 1/[A – A_0_] versus 1/[AcO^−^]^2^ confirming a 1:2 binding ratio between **R1** and AcO^−^ ion as represented in Figure 6. The necessity of two AcO^―^ ions in the binding process could be justified by the fact that a formation of the dimer [(CH_3_COO)_2_H]^−^ is more favored than the formation of CH_3_COOH alone.

**Figure 7:**
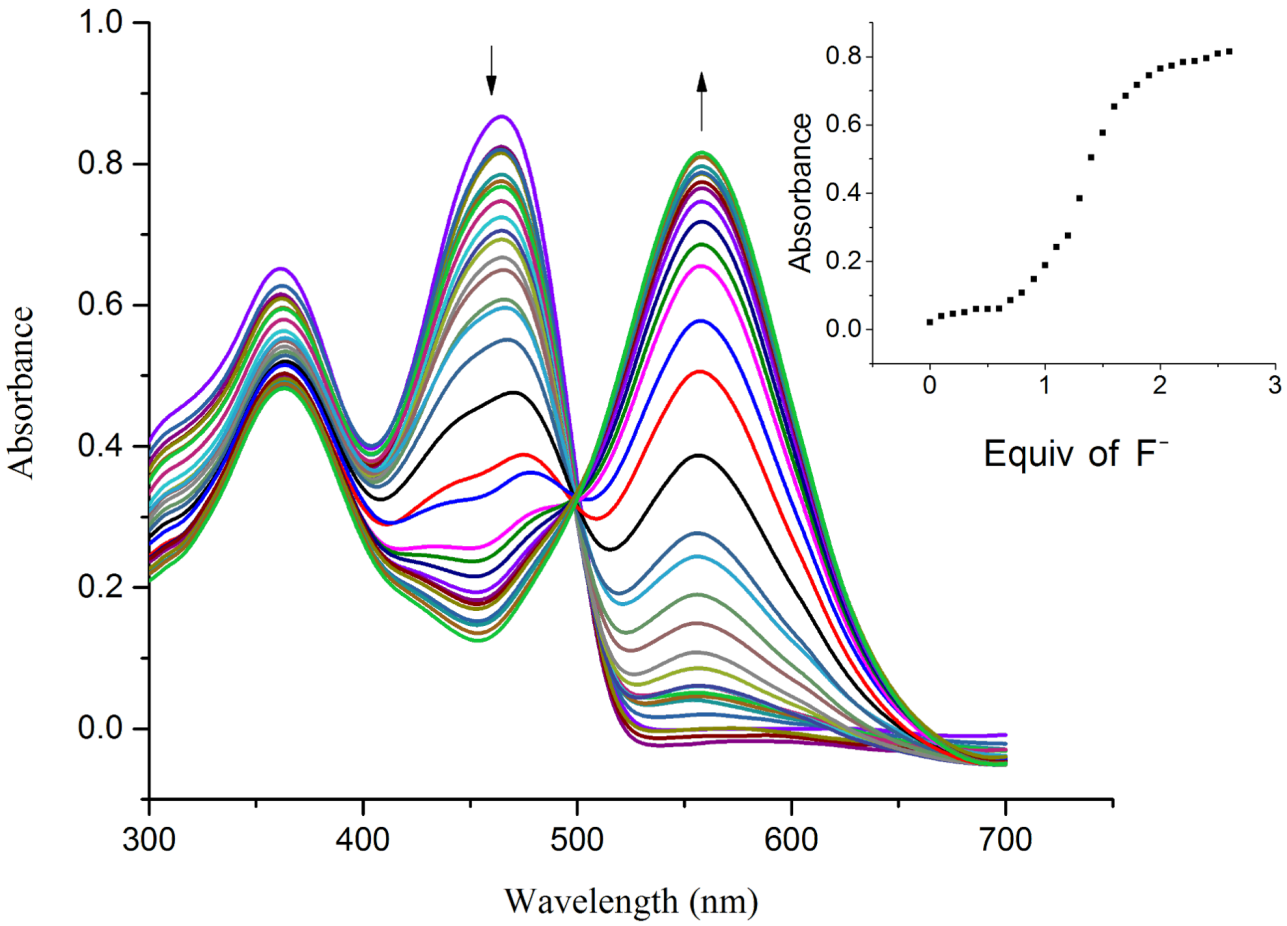
UV–vis titration spectra of receptor **R2** (4.5 × 10^−5^ M in DMSO) obtained by the incremental addition of 0.1 equiv of TBAF (1 × 10^−2^ M in DMSO). The inset plot represents the binding isotherm at 560 nm.

**Figure 8:**
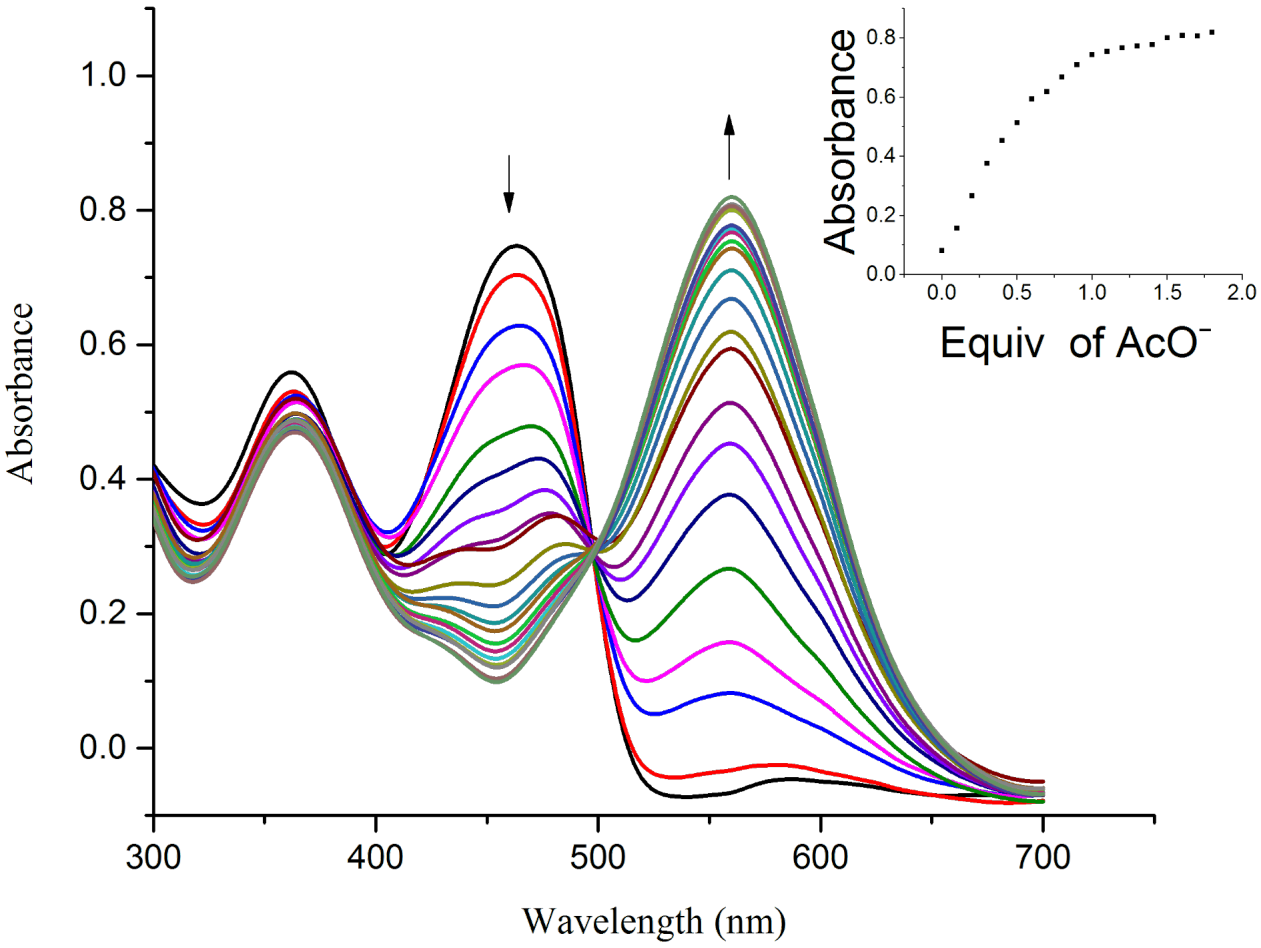
UV–vis titration spectra of receptor **R2** (4.5 × 10^–5^ M in DMSO) obtained by the incremental addition of 0.1 equiv of TBAOAc (1 × 10^−2^ M in DMSO). The inset plot represents the binding isotherm at 560 nm.

**Figure 9:**
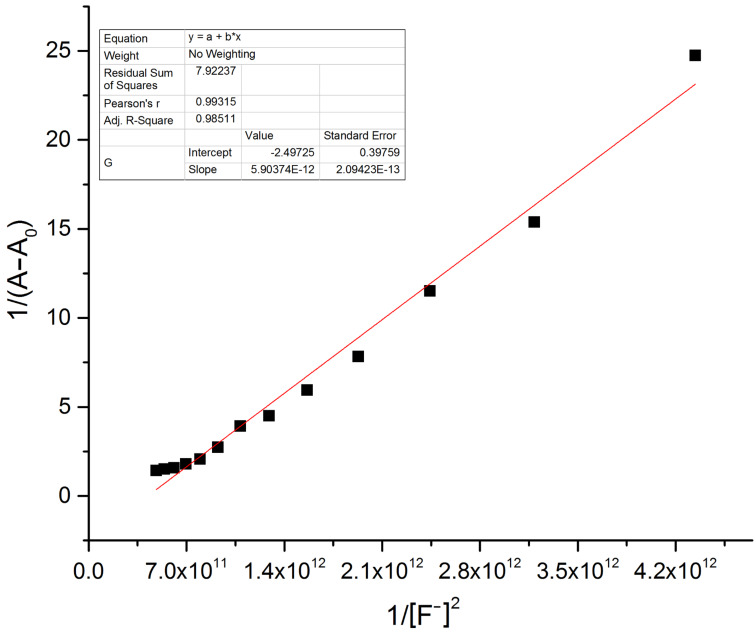
B–H plot of the **R2**–F^−^ complex at a selected wavelength of 560 nm.

**Figure 10:**
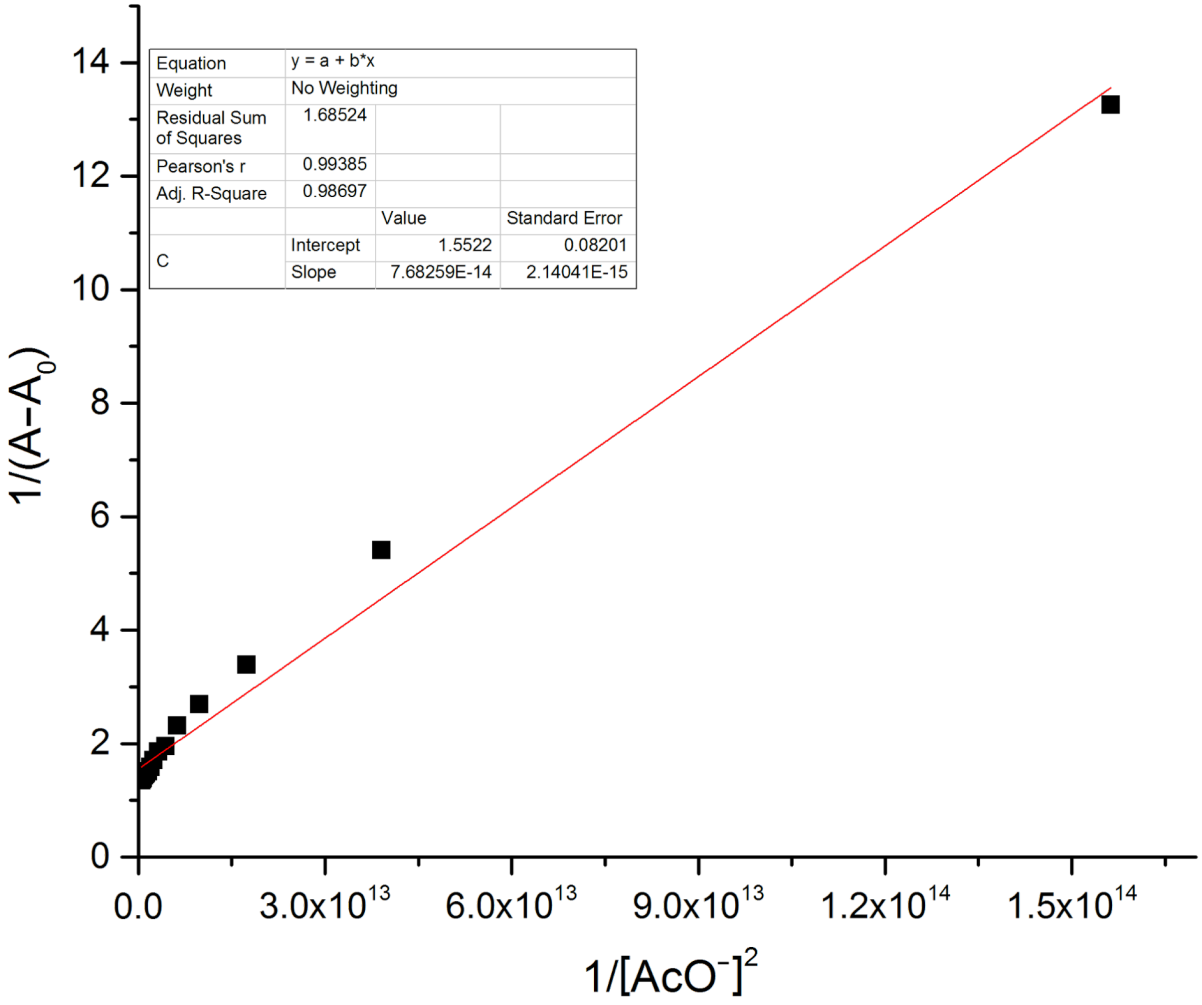
B–H plot of the **R2**–AcO^−^ complex at a selected wavelength of 560 nm.

The UV–vis spectrum of receptor **R2** exhibits an absorption band at 363 nm corresponding to transitions of the azomethine group along with a low-energy band at 459 nm relating to the ICT transition from the naphthyl–OH moiety (donor) to the NO_2_-substituent (acceptor) of the pyridine group. Upon incremental addition of 0.1 equiv of F^−^ and AcO^−^ ions to receptor **R2**, the absorption band at 459 nm red shifted to 560 nm indicative of the strong influence of –R and the −I effect of the NO_2_ substituent on the intermolecular proton-transfer process. A substantial enhancement of the ICT in **R2** in comparison with **R1** indicates a more efficient push–pull tendency existing in the host–guest interaction mechanism. The appearance of a clear isosbestic point at 500 nm clearly indicates the formation of the new complex. The complete disappearance of the absorbance at 459 nm at higher concentrations of fluoride and acetate ions is suggestive of the deprotonation mechanism. The saturation point was achieved with the addition of 2 equiv of the anions indicating the completion of reaction. The titration profile of **R2** with F^−^ and AcO^−^ ions is represented in Figure 7 and Figure 8. The B–H plot for the **R2**–F^−^ and **R2**–AcO^−^ complexes yielded a linear plot with second power of concentration of the anions indicating the strong hydrogen-bond formation followed by deprotonation of the receptor. The B–H plots of **R2** with F^−^ and AcO^−^ ions are shown in Figure 9 and Figure 10.

Fluoride and acetate ions are present as their sodium salts at the physiological level. With this in mind, the development of sensors which can detect anions in aqueous media is of special interest. Thus, titration studies of **R1** (4.5 × 10^−5^ M, DMSO/H_2_O 9:1, v/v) with incremental addition of 0.1 equiv of sodium salts of F^−^ and AcO^−^ ions (10^−2^ M in distilled H_2_O) were performed. A red shift of the band at 395 nm to 473 and 489 nm, respectively, was observed depicting the formation of receptor–anion complexes with clear isosbestic points as shown in Figures S9 and S10 in Supporting Information File 1. The B–H plots corresponding to the **R2**–F^−^ and **R2**–AcO^−^ complex are displayed in Figures S11 and S12 (Supporting Information File 1). The titration experiments of **R2** with sodium salts of F^−^ and AcO^−^ ions revealed redshifts to 556 nm and 559 nm correspondingly with clear isosbestic points indicating complex formation. The titration profiles of **R2** obtained by the addition of sodium fluoride and sodium acetate are shown in Figure S13 and Figure S14 in Supporting Information File 1. Further, stoichiometric ratios of 1:2 of complexes **R2**–F^−^ and **R2**–AcO^−^ are also represented in Supporting Information File 1, Figures S15 and S16, respectively. The resulting binding constants calculated with the B–H equation and the detection limit for **R1** and **R2** are collected in Table 1.

**Table 1:** Calculation of binding constants *K* and detection limit (LOD).

Receptor	Salts	Binding constant (*K*)	LOD (ppm)

**R1**	TBAF	6.8 × 10^1^ M^−1^	9.41
	TBAAcO	2.07 × 10^4^ M^−2^	5.08
	NaF	3.9 × 10^1^ M^−1^	1.71
	NaAcO	1.82 × 10^4^ M^−2^	**1.84**
**R2**	TBAF	0.30 × 10^4^ M^−2^	5.2
	TBAAcO	5.6 × 10^4^ M^−2^	3.39
	NaF	0.85 × 10^4^ M^−2^	**0.94**
	NaAcO	6.3 × 10^4^ M^−2^	**0.92**

**Figure 11:**
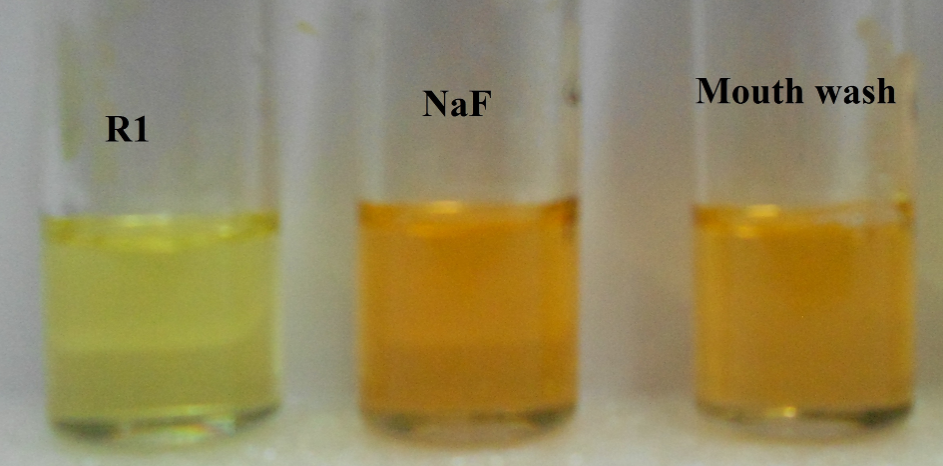
Color change of receptor **R1** upon the addition of NaF and mouthwash.

**Figure 12:**
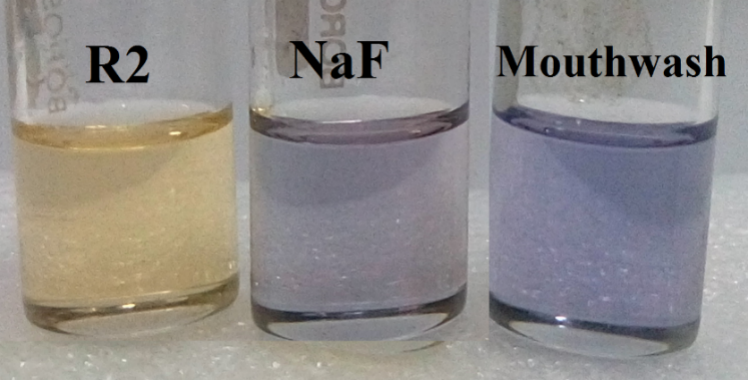
Color change of receptor **R2** upon the addition of NaF and mouthwash.

The ability to detect anions in aqueous media reflects the suppression of solvent interferences in the detection process. Anion binding studies of **R1** and **R2** were extended to the detection of fluoride ions in commercially available mouthwash. Both, **R1** and **R2** exhibited a remarkable color change from pale yellow to orange and blue, respectively, upon the addition of 2 equiv of mouthwash. The color change observed is shown in Figure 11 and Figure 12.

### Time dependency studies

**Figure 13:**
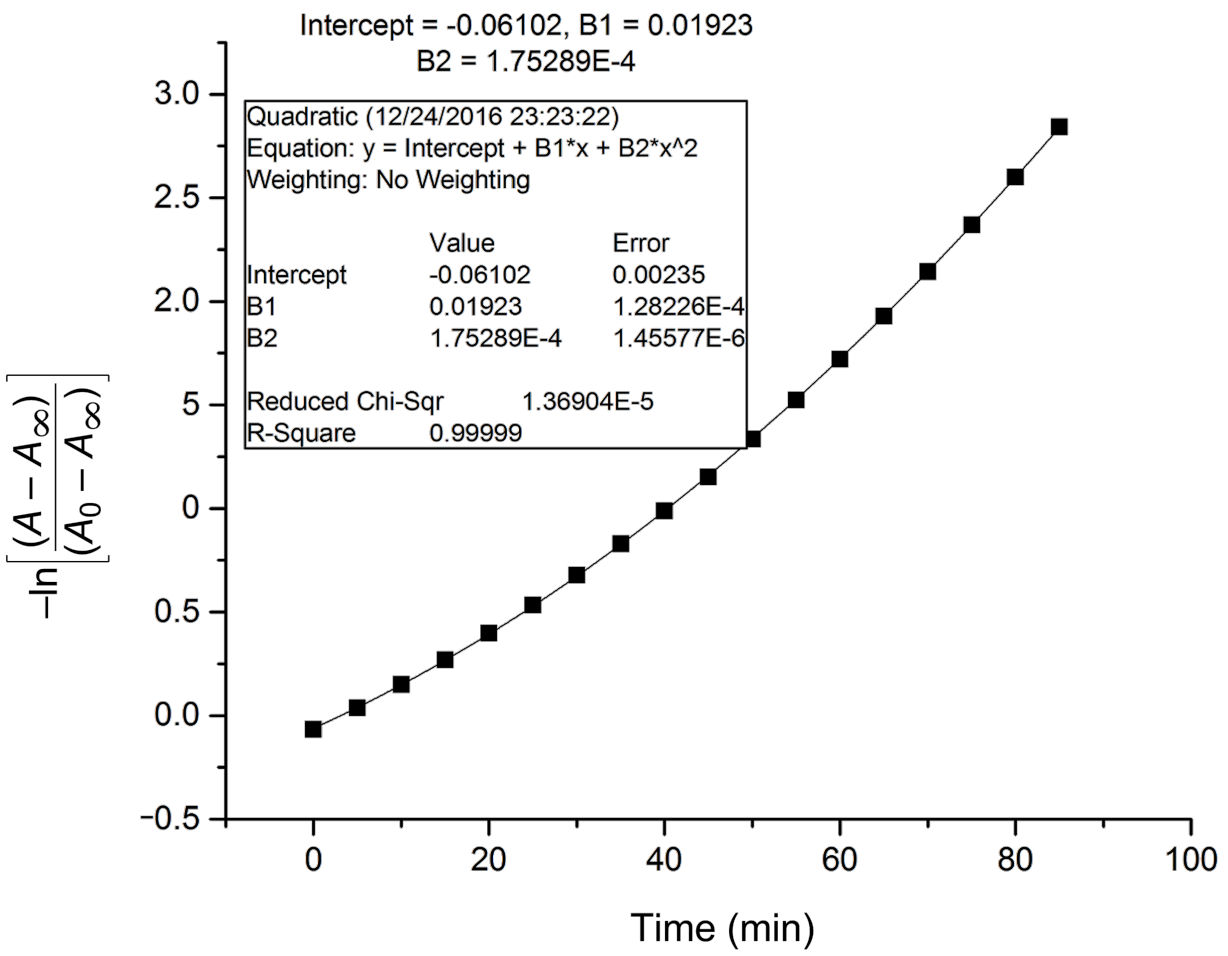
Time dependent plot of first order rate equation to determine the rate constant from the UV–vis spectral change of **R1** in the presence of AcO^−^ ion at 492 nm.

**Figure 14:**
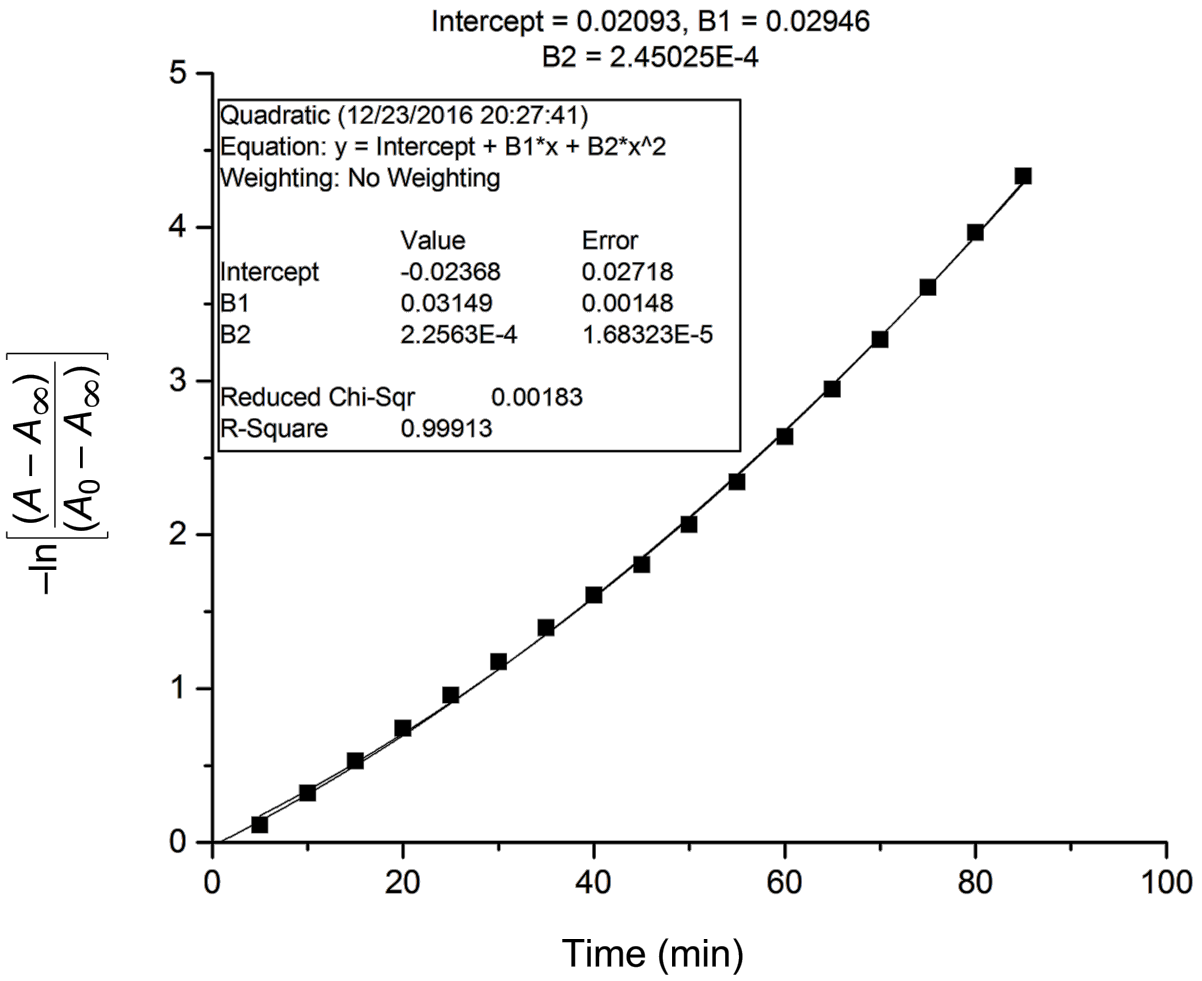
Time dependent plot of first order rate equation to determine the rate constant from the UV–vis spectral change of **R2** in the presence of AcO^−^ ion at 560 nm.

The colorimetric response of receptor **R1** and **R2** towards acetate ions was found to be instantaneous. During the titration experiments, it was observed that with the increase of concentration of AcO^−^ ions, the spectral changes exhibited a substantial increase in the absorption value corresponding to a linear dependency on concentration. The decrease in the intensity of the original absorption bands of receptors **R1** and **R2** centered at 395 nm and 459 nm, respectively, and the gradual increase of the bands centered at 492 nm and 560 nm with clear isosbestic points indicates the complexation process. It is assumed that the AcO^−^ ions interacted with the receptors forming an intermediate compound which further transformed into a stable complex. Further it was observed that the anion binding attained saturation at an anion concentration of 2 equiv after 10 minutes beyond this time there was no significant alteration of the intensity of the absorption band. With this in view, the spectral changes of receptors **R1** and **R2** have been recorded as a function of time with the incremental addition of AcO^−^ ions. Owing to the sharp changes in the UV–vis titration spectra with clear isosbestic points, it could be assumed that there were no significant side reactions. Consequently, we tried to fit the data of the change in absorbance as a function of time to the first order rate equation ln│A − A_∞_│= −*kt* + ln│A_0_ − A_∞_│where A_0_ is the initial absorbance (*t* = 0 min), A as the absorbance at an intermediate (*t* = 5 min) and A_∞_ as the absorbance at saturation (*t* = 10 min) [40]. The rate constant was calculated for **R1** and **R2** as a comparison over the reactivity of receptors towards acetate ions. The rate constant was calculated at two different wavelengths corresponding to the original absorption band of the free receptors and the red-shifted bands observed in the presence of the anion. The time response for AcO^−^ ion monitoring the band at 492 nm and 560 nm for **R1** and **R2**, respectively, is shown in Figure 13 and Figure 14. The rate constants calculated for the band at 395 (**R1**) and 492 (**R1** + AcO^−^); 459 nm (**R2**) and 560 nm (**R2** + AcO^−^) are too close indicating the relative dependence of the anion concentration on the reacting species. Similarly, the rate constants have been calculated for **R1** and **R2** in the presence of fluoride. The lower order of magnitude of the rate constant in the presence of F^−^ ions could be correlated to the p*K*_a_ value of 3.2 (F^−^ ion) in comparison with AcO^−^ ion whose p*K*_a_ value is 4.8. The time response of receptors **R1** and **R2** in the presence of AcO^−^ ion is represented in Supporting Information File 1, Figures S17 and S18. The observed rate constants at different wavelengths for **R1** and **R2** are summarized in Table 2.

**Table 2:** Observed rate constants for the reaction of receptor **R1** and **R2** with different anions.

Anion	Rate constant *k* (min^−1^)			
	
	**R1**		**R2**	
	395 nm	492 nm	459 nm	560 nm

AcO^−^	0.0019	0.0018	0.00216	0.0027
F^−^	0.0000069	0.000078	0.00005	0.00014

### ^1^H NMR titration studies

**Figure 15:**
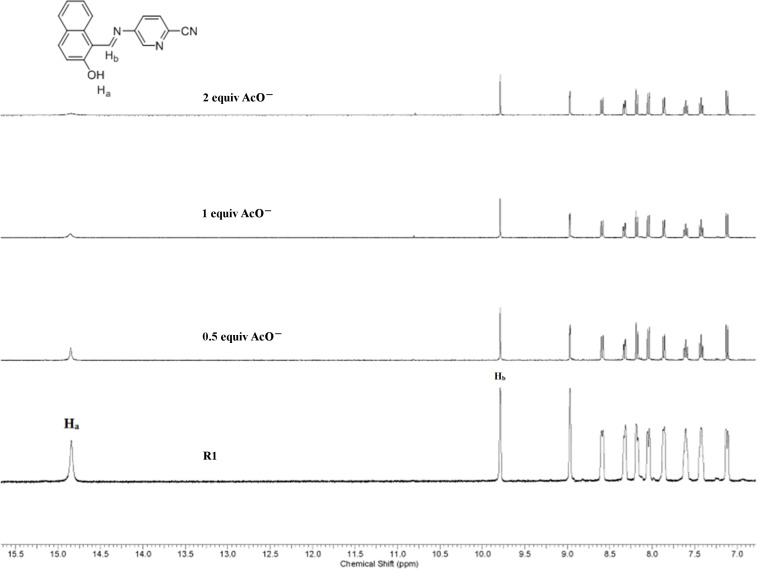
^1^H NMR titration spectra of **R1** upon incremental addition of AcO^−^ ion.

**Figure 16:**
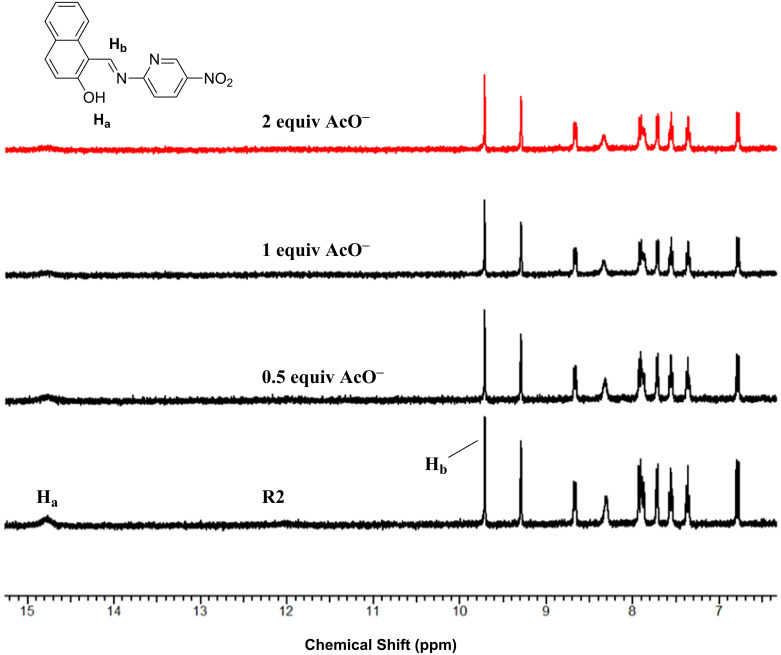
^1^H NMR titration spectra of **R2** upon incremental addition of AcO^−^ ion.

To gain insight into the binding mechanism, ^1^H NMR titration studies have been performed with the incremental addition of TBAOAc to a DMSO-*d*_6_ solution of receptor **R1** and **R2**. The unbound receptor exhibited a OH proton signal at ~14 ppm due to the presence of an intramolecular hydrogen bond interaction with the imine nitrogen [41–43]. The proton corresponding to the OH group centered at 14.8 ppm (**R1**) and 14.78 ppm (**R2**) exhibited a strong hydrogen bond with AcO^−^ ion indicated by the signal broadening upon successive addition of 0.5 and 1 equiv of the anion. In the presence of 2 equiv AcO^−^ ion, the proton signal diminished indicating deprotonation. The imine proton did not exhibit an upfield or downfield shift, yet the signal intensity decreased upon successive addition indicative of its involvement in the bifurcated hydrogen bond interaction with the AcO^−^ ion. The aromatic protons in **R1** and **R2** exhibited a gradual decrease in intensity indicating the charge-transfer interactions occurring in the presence of the AcO^−^ ion. The ^1^H NMR titration spectra of **R1** and **R2** in the presence of increasing concentrations of the AcO^−^ ion are shown in Figure 15 and Figure 16.

### Theoretical studies

In order to understand the binding mechanism, we have performed a density functional theory (DFT) simulation on the receptor molecules using the GAUSSIAN 09 software package [44]. A closed-shell Becke–Lee–Yang–Parr hybrid exchange–correlation three-parameter functional (B3LYP) [45] along with the 6-311++G(d,p) basis set were used in the simulation to derive a complete geometry optimization for the isolated receptors as well as the receptor binding with AcO^−^ and F^−^ ions. The basis set 6-311++G (d,p) augmented by ‘d’ polarization functions on heavy atoms and ‘p’ polarization functions on hydrogen atoms was used [46–47]. The molecular geometry was fully optimized by using Berny’s optimization algorithm, which uses redundant internal coordinates. Molecular orbitals (HOMO, LUMO) were plotted using the GaussView software. In a second step the time-dependent DFT (TD-DFT) method was used considering the same B3LYP exchange–correlation functional with the 6-311++G (d,p) basis set to obtain the UV–vis absorption spectra of the free and ion-bonded receptor in DMSO as the solvent.

**Figure 17:**
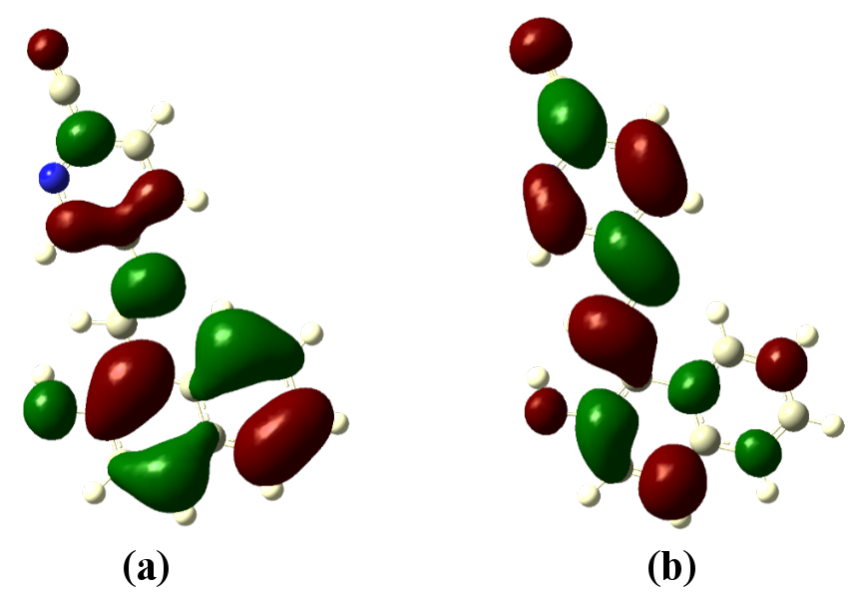
Optimized structure of receptor **R1**; (a) HOMO, (b) LUMO.

**Figure 18:**
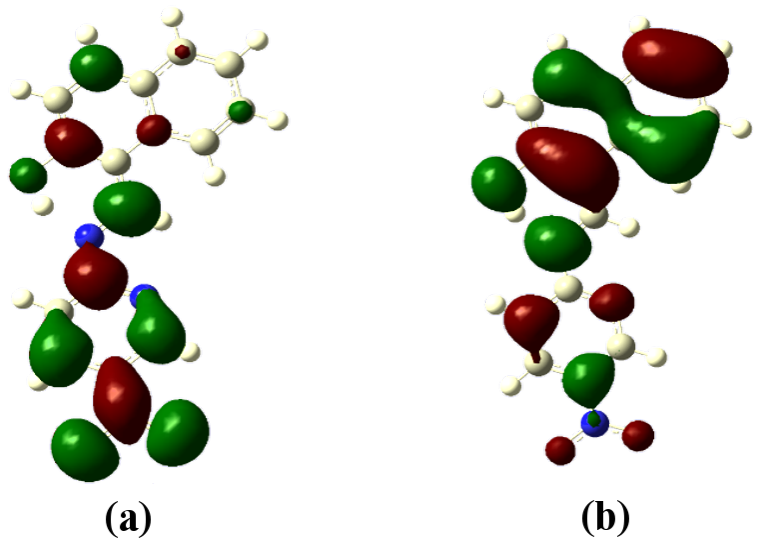
Optimized structure of the receptor **R2**; (a) HOMO, (b) LUMO.

**Figure 19:**
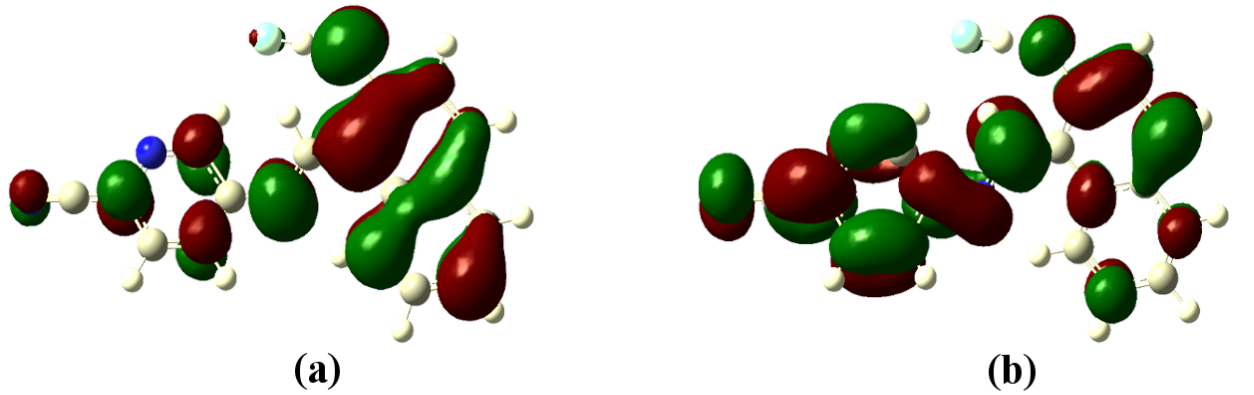
Optimized structure of the **R1**-F^−^ complex; (a) HOMO, (b) LUMO.

**Figure 20:**
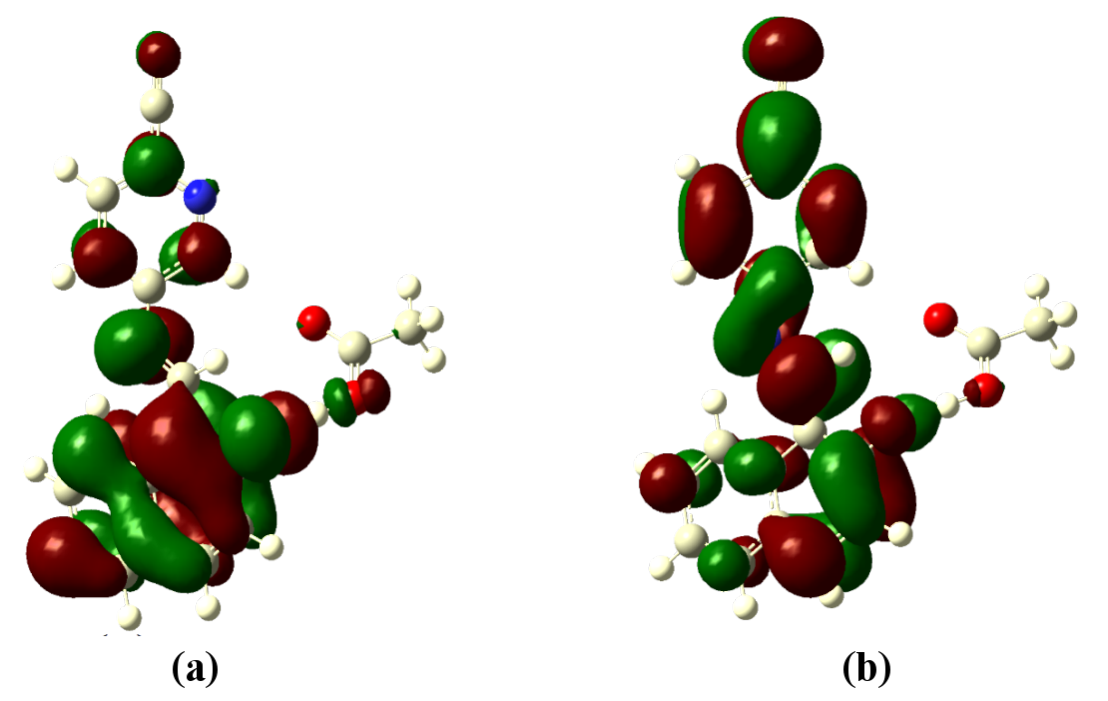
Optimized structure of the **R1**-AcO^−^ complex; (a) HOMO, (b) LUMO.

**Figure 21:**
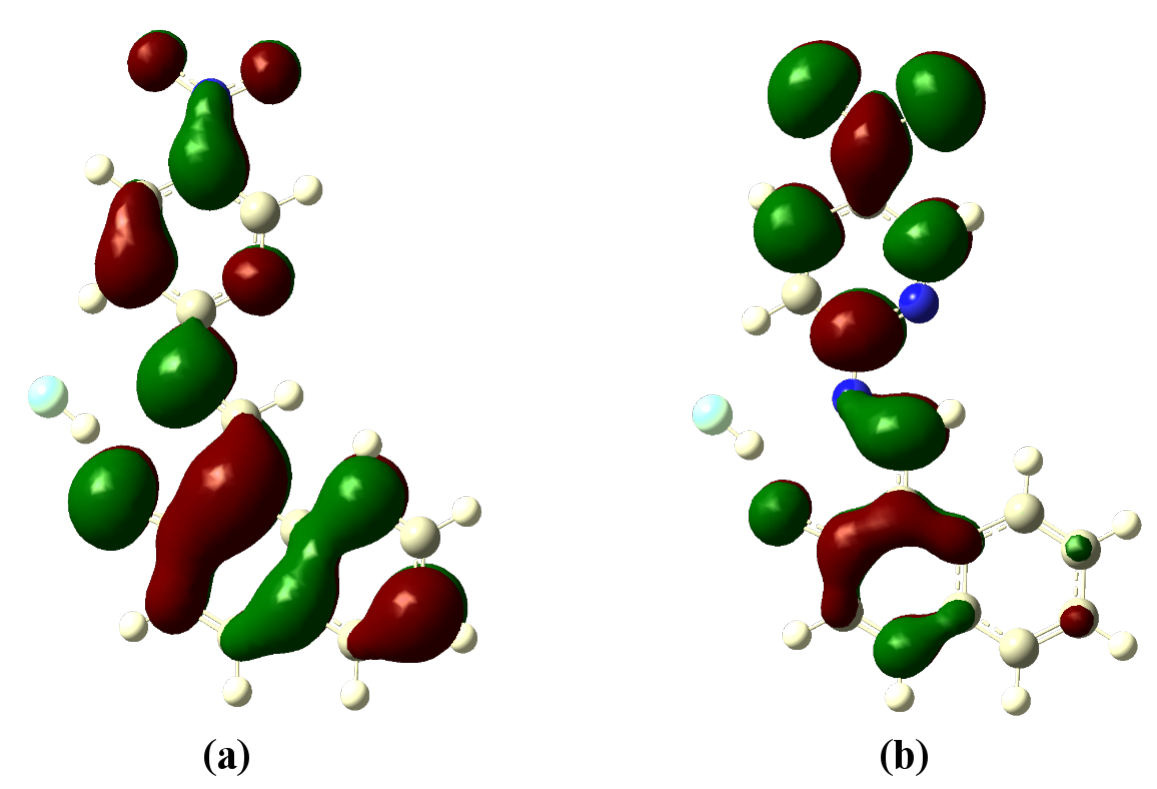
Optimized structure of the **R2**-F^−^ complex; (a) HOMO, (b) LUMO.

**Figure 22:**
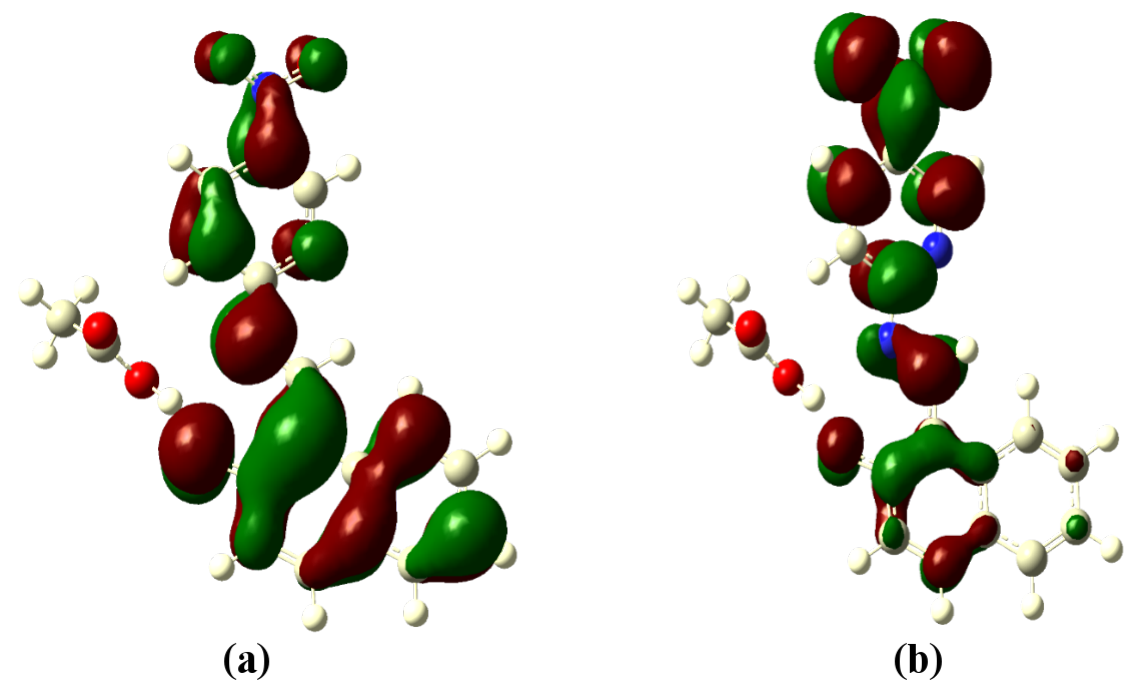
Optimized structure of the **R2**-AcO^−^ complex; (a) HOMO, (b) LUMO.

The optimized structures of the receptors **R1** and **R2** with the distribution of their HOMO and LUMO levels are represented in Figure 17 and Figure 18. It was found that there were no conformational changes observed in receptors **R1** and **R2** in the presence of anions indicating the structural stability of the receptor–anion complex. The results show that the HOMOs and LUMOs are spread over both aromatic rings due to the presence of the electron-withdrawing nitro and cyano functionalities. The energy differences Δ*E* which correspond to the energy difference between HOMO and LUMO (*E*_HOMO_ − *E*_LUMO_) were calculated for **R1** and **R2** and found to be 0.1252 *E*_h_ and 0.09 *E*_h_, respectively. In order to confirm the stability of receptor–anion complex, the HOMOs and LUMOs in the presence of F^−^ and AcO^−^ ions were also studied. A significant reduction of Δ*E* to 0.0679 Ha (**R1** + F^−^), 0.04 (**R1** + AcO^−^) and 0.08 Ha (**R2** + F^−^ and **R2** + AcO^−^) confirms the presence of intramolecular charge-transfer transitions during the anion detection process. The similar values of Δ*E* observed with **R2** in the presence of F^−^ and AcO^−^ ions is attributed to the nearly identical absorption maxima (560 nm) obtained for **R2**–F^−^ and **R2**–AcO^−^ complexes. The reduction of the band gap values is supported with a red shift of the original absorption band of the receptors **R1** and **R2**. The emergence of a new band at higher wavelength confirms the complex formation process. The HOMOs and LUMOs of **R1**–F^−^ and **R1**–AcO^−^ are represented in Figure 19 and Figure 20; the HOMOs and LUMOs of **R2**–F^−^ and **R2**–AcO^−^ are represented in Figure 21 and Figure 22. The decrease in the bond length value corresponding to the OH group from 0.96 Å to 1.47 Å and 1.55 Å reflects the host–guest interaction. Mulliken charge distribution calculations showed a change of the atomic charge on the oxygen atom of receptors **R1** and **R2** from less negative to more negative values which are indicative for an intramolecular charge transfer process upon anion binding (Supporting Information File 1, Table S1). Theoretical calculations afforded absorption maxima at 352 nm and 416 nm for receptor **R1** and 361 and 487 nm for **R2**, respectively. The shift in the absorption maxima for **R1**–F^−^ and **R1**–AcO^−^ to 451 and 455 nm and for **R2**–F^−^ and **R2**–AcO^−^ to 572 and 571 nm fully supports the anion-binding process. A substantial increase in the dipole moment of the fluoride complex indicates an efficient charge separation allowing the formation of the hydrogen bond between OH and F^−^. While the corresponding acetate complex exhibited a two-fold increase in the dipole moment implying the bifurcated nature of the hydrogen bond involving one OH proton with two electronegative oxygen atoms of the acetate ion.

### Binding mechanism

**Scheme 1:**
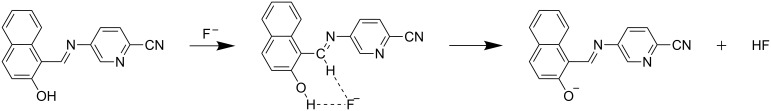
Proposed binding mechanism of **R1** with fluoride ion.

**Scheme 2:**
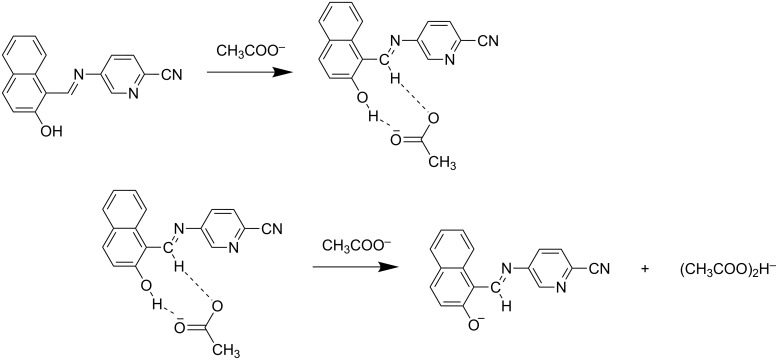
Proposed binding mechanism of **R1** with acetate ion.

**Scheme 3:**
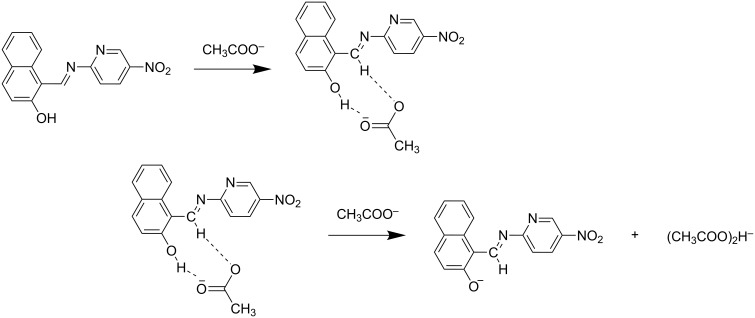
Possible binding mechanism of **R2** with acetate ion.

**Scheme 4:**
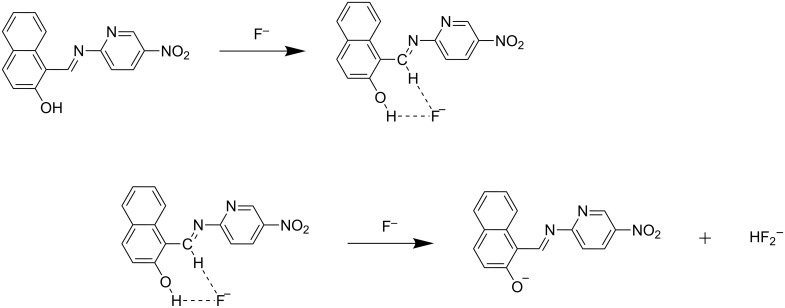
Proposed binding mechanism of **R2** with fluoride ion.

Based on the ^1^H NMR titration studies the following binding mechanism is proposed. The receptors **R1** and **R2** undergo an anion-induced deprotonation of the OH group during incremental addition of F^−^ and AcO^−^ ions. The binding mode of **R1** with fluoride and acetate is represented in Scheme 1 and Scheme 2. The binding of **R2** with F^−^ and AcO^−^ ion is represented in Scheme 3 and Scheme 4.

### Logic gate applications

We checked the colorimetric response of receptors **R1** and **R2** (4.5 × 10^−5^ M in DMSO) towards the cations Na^+^, K^+^, Ca^2+^, Mg^2+^, Al^3+^, Co^2+^, Ni^2+^, Cu^2+^, Zn^2+^, Pb^2+^, Cd^2+^ and Hg^2+^ that were used as nitrate salts at a concentration of 10^−3^ M in distilled water. A strong colorimetric response of receptor **R1** towards Hg^2+^ cations resulting in a color change from pale yellow to colorless occurred together with a decrease in the absorption band centered at 395 nm. The binding of cations to receptor **R1** follows the hard soft acid base concept. Further a charge transition from the ligand to the metal allows the selective binding of Hg^2+^ ion accompanied by the aforementioned color change. The stepwise addition of Hg^2+^ ions to a solution of **R1** leads to an increased intensity and bathochromic shift by 8 units of the absorption band at 266 nm. On the other hand the intensities of the bands centered at 325 nm and 395 nm decreased due to the deprotonation of the hydroxy group. An isosbestic point is observed at 323 nm representing the interaction of Hg^2+^ with **R1** involving the imine group and the oxygen of the hydroxy group. The corresponding B–H plot displayed a 1:1 complexation ratio between **R1** and Hg^2+^ ion. A picture of the observed color change and the corresponding UV–vis spectra are shown in Supporting Information File 1, Figures S19 and S20. There was no remarkable colorimetric response in the presence of the other cations tested in the current study.

**Figure 23:**
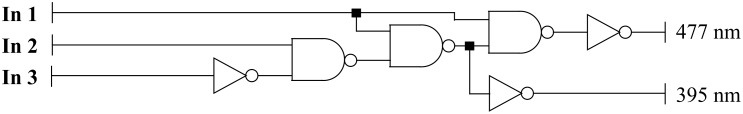
Logic circuit for the “INHIBIT” gate of receptor **R1**.

In case of receptor **R2** no color change was observed in the presence of the cations implying its role as an anion sensor. As receptor **R1** behaved as a dual sensor being sensitive towards fluoride and acetate anions and Hg^2+^ as cation, we have fabricated logic gate circuits and performed arithmetic calculations at molecular level [48]. The dual ion sensing property of **R1** has been extended to develop a logic circuit constituting “INHIBIT” functions at the molecular level using F^−^ and Hg^2+^ ions as chemical input. The UV–vis spectrum of receptor **R1** (4.5 × 10^−5^ M in DMSO) in the presence of fluoride as anion resulted in an output at 477 nm exhibiting a simple “YES” response. In the presence of Hg^2+^ as third input (In 3), the output signal corresponding to the ‘ON’ state (HIGH) at 477 nm is minimized. According to arithmetic calculations, this could be considered as ‘OFF’ state (0) [48–51]. The ‘OFF’ state is likely due to the formation of HgF_2_ which is responsible for the retrieval of receptor **R1** in its original form. A significant hyperchromic effect is observed for the band at 477 nm when In 2 (F^−^) is in the ‘ON’ mode, i.e., during the **R1**–F^−^ complex formation process. With the introduction of Hg^2+^ (In 3), the output attains the zero state (‘OFF’). In total, three input combinations lead to an “INHIBIT” logic gate. The “INHIBIT” logic gate and truth table is represented in Figure 23 and Table 3.

**Table 3:** "INHIBIT" logic gate for receptor **R1** at 477 nm

In 1	In 2	In 3	395 nm	477 nm

1	0	0	1	0
1	0	1	1	0
1	1	0	0	1
1	1	1	1	0

## Conclusion

Two new organic receptors exhibiting a positional substitution effect have been designed, synthesized and characterized. They were shown to allow the qualitative and quantitative detection of anions without the need of expensive equipment and sophisticated instrumentation. UV–vis titration experiments confirmed a modulation of the donor hydrogen bond ability as a direct consequence of the different substituents attached to the aromatic ring leading to a shift of the absorption maxima during the binding process. Receptors **R1** and **R2** showed a higher selectivity towards AcO^−^ ions in organic and aqueous media owing to its shape complementarity and relatively high basicity compared to other anions. A practical application of the colorimetric responses of **R1** and **R2** as real time sensors towards fluoride ions was demonstrated in commercially available mouthwash. The ability of **R1** and **R2** to exhibit colorimetric responses to fluoride ions present in commercially available mouthwash demonstrated their practical utility as real time sensors. Based on determined binding constants, the order of reactivity of receptors towards suitable anions was found to be **R2** > **R1**. The low detection limit of 1.84 ppm and 0.92 ppm for sodium acetate reflect the high sensitivity of receptors **R1** and **R2** in the anion detection surpassing the constraints of aqueous media. Time dependency studies confirmed a first order rate equation for the anion binding process. ^1^H NMR and TD-DFT calculations further confirmed the anion binding process of receptor **R1** and **R2** with F^−^ and AcO^−^ ions. The detection ability of Hg^2+^ and F^−^ ions by receptor **R1** allows its usage in molecular logic gate applications.

## Experimental

### Materials and methods

All chemicals used in the present study were procured from Sigma-Aldrich, Alfa Aesar or Spectrochem and used as received. All solvents were purchased from SD Fine, India, were of HPLC grade and used without further distillation. Melting points were measured on a Stuart SMP3 melting point apparatus in open capillaries. Infrared spectra were recorded on a Bruker alpha FTIR spectrometer. UV–vis spectroscopy was performed with an Analytik Jena Specord S600 spectrometer in a standard 3.0 mL quartz cell with 1 cm path length. The ^1^H NMR spectra were recorded on Bruker Ascend (400 MHz) instrument using TMS as internal reference and DMSO-*d*_6_ as solvent. Resonance multiplicities are described as s (singlet), d (doublet), t (triplet) and m (multiplet). Mass spectra were recorded using a DART-MS, JMS-T100LC, Accu TOF Mass Spectrometer. Elemental analysis was performed with an ELEMENTOR Micro analysis system, varioMICRO CUBE CHNS.

**Scheme 5:**
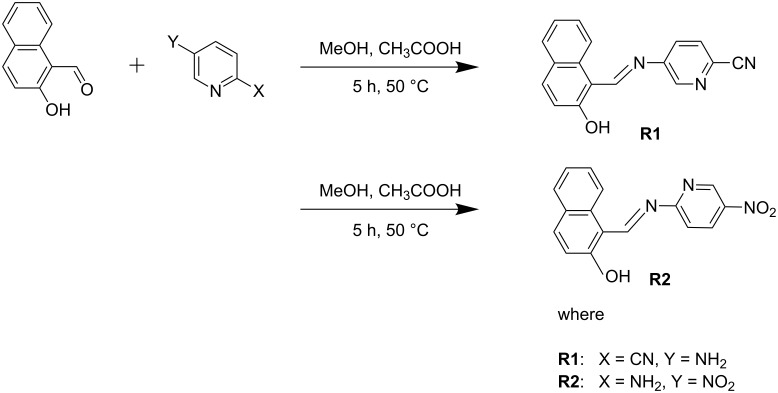
General scheme for the synthesis of receptors **R1** and **R2**.

**Synthesis of receptors R1 and R2:** Receptors **R1** and **R2** were synthesized through a Schiff base condensation reaction of the corresponding aromatic aldehyde and amine (Scheme 5).

### Synthesis of (*E*)-5-(((2-hydroxynaphthalen-1-yl)methylene)amino)picolinonitrile (**R1**)

2-Cyano-5-aminopyridine (0.1 g, 0.83 mmol) and 2-hydroxynaphthaldehyde (0.14 g, 0.83 mmol) were appropriately weighed and transferred into a round-bottomed flask. Methanol (5 mL) and a drop of acetic acid were added and the mixture was heated for about 5 h at 50 °C. The progress of the reaction was followed by TLC. After cooling to rt, the reaction mixture was filtered through a paper filter and washed with methanol to obtain the pure product. Yield: 93%; mp 248 °C; FTIR (cm^−1^): 1545 (ring stretch), 1614 (C=N stretch), 2220 (C≡N), 2978 (=C-H), 3745 (OH stretch); ^1^H NMR (400 MHz, DMSO-*d*_6_, ppm) δ 14.8 (s, OH), 9.79 (s, CH=N), 8.96 (s, Ar-H), 8.5 (dd, Ar-H), 8.31 (d, Ar-H), 8.1 (d, Ar-H), 8.0 (d, Ar-H), 7.85 (d, Ar-H), 7.60 (d, Ar-H), 7.42 (d, Ar-H), 7.1 (dd, Ar-H); MS (*m*/*z*): calcd 273.09; found [M + H]^+^ 274.1; Anal. calcd for C_17_H_11_N_3_O: C, 74.71; H, 4.06; N, 15.38; O, 5.85; found: C, 74.74; H, 3.994; N, 15.4; O, 5.83.

### Synthesis of (*E*)-1-(((5-nitropyridine-2-yl)imino)methyl)naphthalen-2-ol (**R2**)

2-Amino-5-nitropyridine (0.1 g, 0.71 mmol) and 2-hydroxynaphthaldehyde (0.12 g, 0.71 mmol) were appropriately weighed and transferred into a round-bottomed flask. Methanol (5 mL) and a drop of acetic acid were added and the mixture was heated for about 5 h at 50 °C. The progress of the reaction was monitored by TLC. After cooling to rt, the reaction mixture was filtered through a paper filter and washed with methanol to obtain the pure product. Yield: 78%; mp 227 °C; FTIR (cm^−1^): 1545 (ring stretch), 1630 (C=N stretch), 2978 (C=N), (=C-H), 3364 (Ar CH), 3494 (OH stretch); ^1^H NMR (400 MHz, DMSO-*d*_6_, ppm) δ 14.78 (s, OH), 9.71 (s, CH=N), 9.29 (s, Ar-H), 8.66 (d, Ar-H), 8.30 (d, Ar-H), 7.9 (dd, Ar-H), 7.71 (d, Ar-H), 7.73 (d, Ar-H), 7.56 (dd, Ar-H), 7.37 (d, Ar-H), 6.78 (d, Ar-H); MS (*m*/*z*) calcd 293.08; found [M + H]^+^ 294.15; Anal. calcd for C_16_H_11_N_3_O_3_: C, 65.53; H, 3.78; N, 14.33; O, 16.36; found: C, 65.48; H, 3.72; N, 14.19; O, 16.29.

## Supporting Information

File 1 Copies of spectra, B–H plots, B–H equation and Mulliken charge distributions.
