# Supplementary material for: Spectral and DFT studies of anion bound organic receptors: Time dependent studies and logic gate applications
Source: Beilstein J Org Chem. 2017 Feb 6;13:222–38. doi: 10.3762/bjoc.13.25 (PMC5331291; doi:10.3762/bjoc.13.25)
Supplement: File 1 — Copies of spectra, B–H plots, B–H equation and Mulliken charge distributions. [file Beilstein_J_Org_Chem-13-222-s001.pdf]

Supporting Information

for

Spectral and DFT studies of anion bound organic receptors: Time dependent studies and logic gate applications

Srikala Pangannaya[1§], Neethu Padinchare Purayil [1§], Shweta Dabhi[2], Venu Mankad[2], Prafulla K. Jha[3], Satyam Shinde[4], Darshak R. Trivedi[1*]

Address: [1]Supramolecular Chemistry Laboratory, Department of Chemistry, National Institute of Technology Karnataka (NITK), Surathkal, India, [2]Department of Physics, Maharaja Krishnakumarsinhji Bhavnagar University, Bhavnagar 364001, India, [3]Department of Physics, Faculty of Science, The M.S. University of Baroda, Vadodara 390002, India and [4]School of Technology, Pandit Deendayal Petroleum University, Gandhinagar 382007, Gujarat, India

Email: Darshak R. Trivedi - darshak_rtrivedi@yahoo.co.in

[§]equally contributing authors

[$]Tel.: +91-824 2473205; Fax: +91 824 247033

*Corresponding author

Copies of spectra, B–H plots, B–H equation and Mulliken charge distributions

Contents

Figure S1: FTIR spectrum of receptor **R1**
Figure S2: ^{1}H NMR spectrum of receptor **R1**
Figure S3: Mass spectrum of receptor **R1**
Figure S4: FTIR spectrum of receptor **R2**
Figure S5: ^{1}H NMR spectrum of receptor **R2**
Figure S6: Mass spectrum of receptor **R2**
Figure S7: UV–vis absorption spectra of **R1** (4.5×10^{-5} M in DMSO) upon addition of 1 equiv of various anions as TBA salts
Figure S8: UV–vis absorption spectra of **R2** (4.5×10^{-5} M in DMSO) upon addition of 1 equiv of various anions as TBA salts
Figure S9: UV–vis titration spectra of receptor **R1** (4.5×10^{-5} M in DMSO/H_2O 9:1 (v/v)) with the addition of 0.1 equiv of NaF (1×10^{-2} M in distilled water). Inset plot represents the binding isotherm at 473 nm.
Figure S10: UV–vis titration spectra of receptor **R1** (4.5×10^{-5} M in DMSO/H_2O 9:1 (v/v)) with the addition of 0.1 equiv of NaAcO (1×10^{-2} M in distilled water). Inset plot represents the binding isotherm at 489 nm
Figure S11: B–H plot of receptor **R1**-F^- (as NaF) complex at a selected wavelength of 473 nm
Figure S12: B–H plot of receptor **R1**-AcO^- (NaAcO) complex at a selected wavelength of 489 nm
Figure S13: UV–vis titration spectra of receptor **R2** (4.5×10^{-5} M in DMSO/H_2O 9:1 (v/v)) with the addition of 0.1 equiv of NaF (1×10^{-2} M in distilled water). Inset plot represents the binding isotherm at 556 nm
Figure S14: UV–vis titration spectra of receptor **R2** (4.5×10^{-5} M in DMSO/H_2O 9:1 (v/v)) with the addition of 0.1 equiv of NaAcO (1×10^{-2} M in distilled water). Inset plot represents the binding isotherm at 559 nm
Figure S15: B–H plot of receptor **R2**-F^- (NaF) complex at a selected wavelength of 556 nm
Figure S16: B–H plot of receptor **R2**-AcO^- (NaAcO) complex at a selected wavelength of 559 nm
Figure S17: Time dependency plot of first order rate equation to determine the rate constant from UV–vis spectral change of **R1** in the presence of F^- ion at 492 nm

Figure S18: Time dependency plot of first order rate equation to determine the rate constant from UV–vis spectral change of **R2** in the presence of F^- ion at 560 nm
Figure S19: Color change of receptor **R1** (4.5×10^{-5} M in DMSO) upon the addition of 1 equiv of a variety of cations (10^{-3} M in distilled water)
Figure S20: UV–vis absorption spectra of **R1** (4.5×10^{-5} M in DMSO) in the presence of different cations (1×10^{-3} M in distilled water)
Table S1: Mulliken charge distribution of **R1, R1**+F^-, **R1**+AcO^- and **R2**, **R2**+F^-, **R2**+AcO^-
Equation for calculation of binding constant
Equation for calculation of detection limit
References

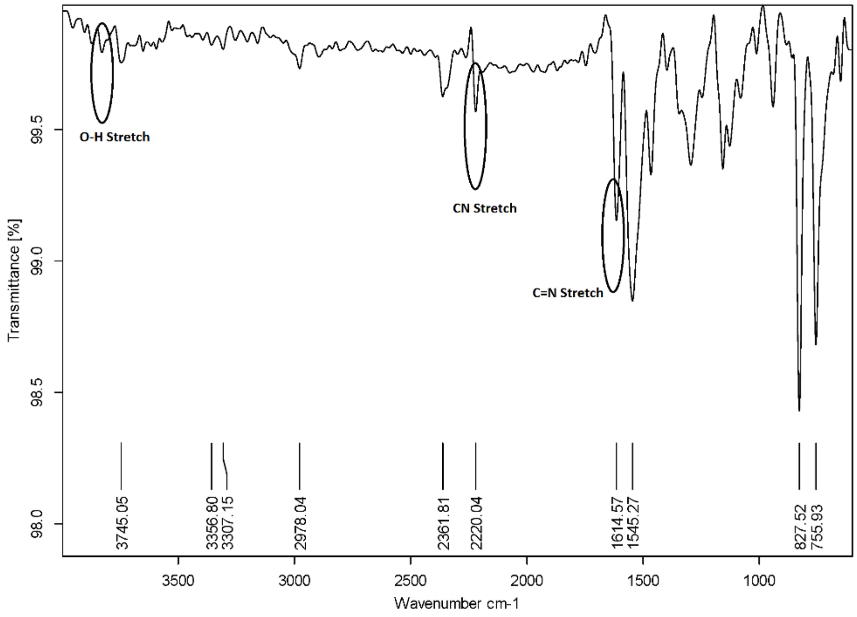

Figure S1: FTIR spectrum of receptor **R1**

FT-IR (cm^{-1}): (ring stretch) 1545, (C=N stretch) 1614, (C≡N) 2220, (=C-H) 2978, (-OH stretch) 3745.

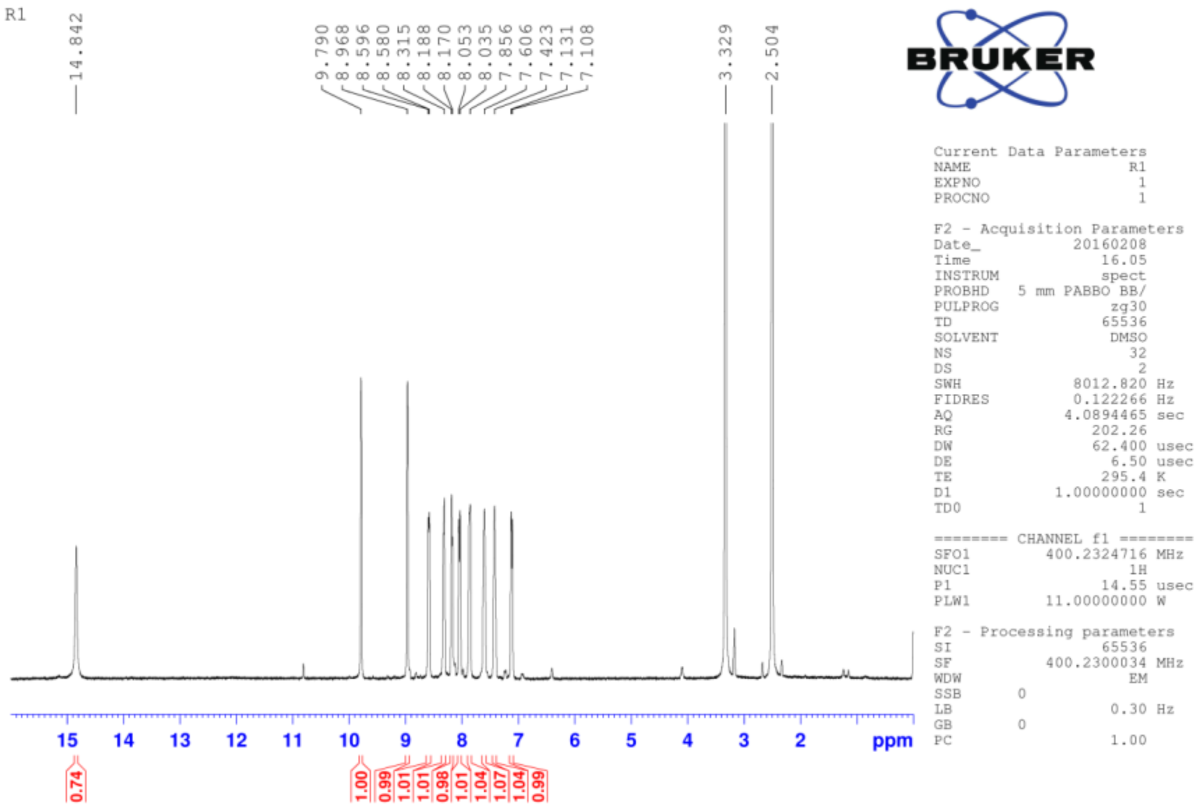

Figure S2: ^{1}H NMR spectrum of receptor **R1**

^{1}H NMR (DMSO-d_6, 400 MHz, ppm): δ 14.8 (s,OH), 9.7 (s, Ar-H), 8.9 (s,CH=N), 8.0(d, Ar-H), 8.1 (d, Ar-H),8.3 (s, Ar-H), 8.5 (d, Ar-H), 7.4 -7.8(s,3Ar-H),7.1(d,Ar-H)

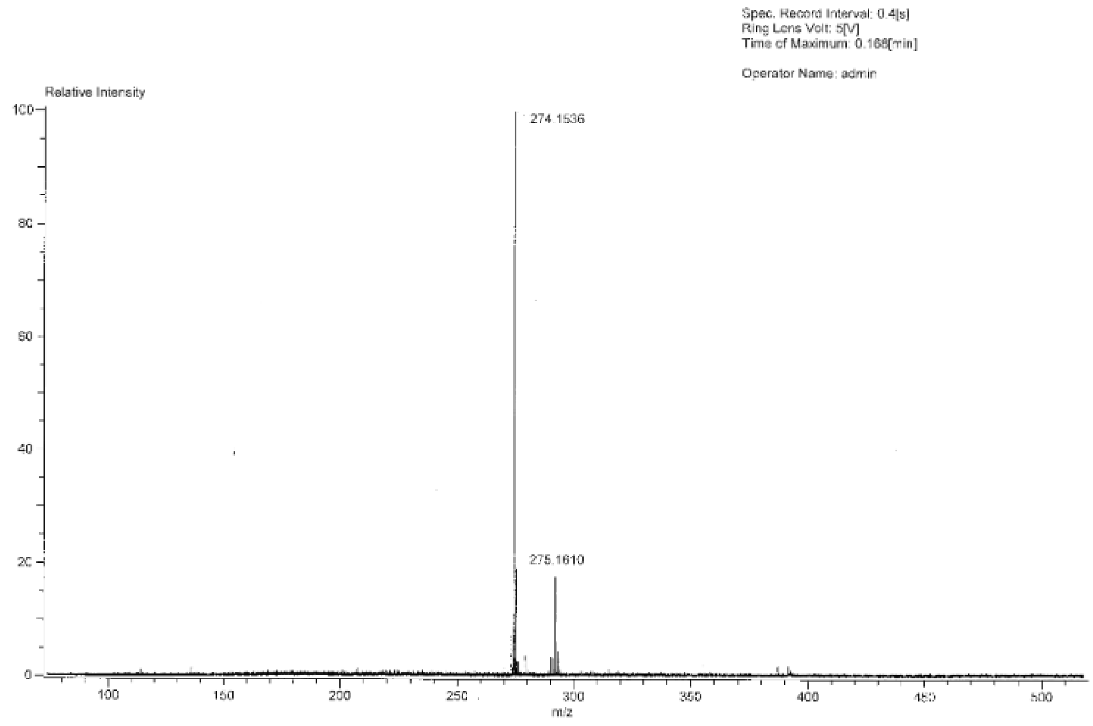

Figure S3: Mass spectrum of receptor **R1**

Calculated: 273.09

Obtained: (M+ H $^{+}$) 274.1

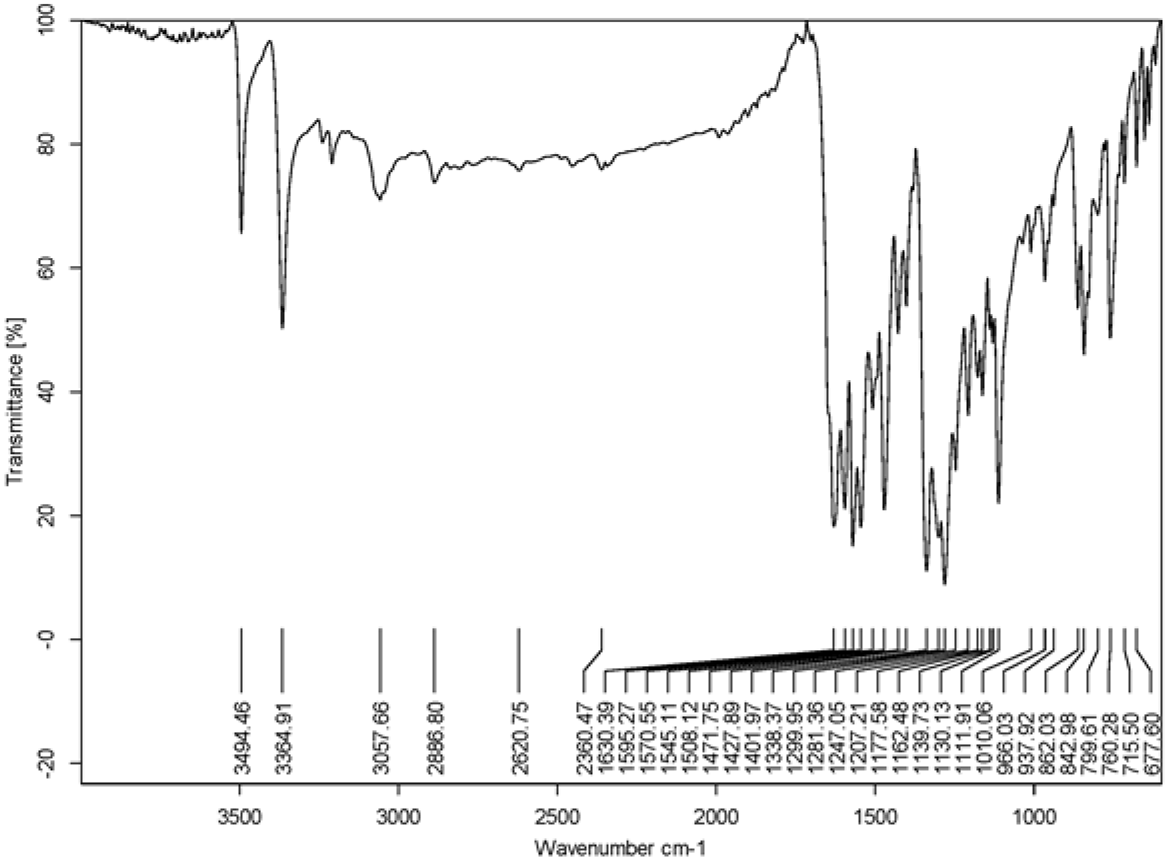

Figure S4: FTIR spectrum of receptor **R2**

FT-IR (cm^{-1}): (ring stretch) 1545, (C=N stretch) 1630, (C=N),(=C-H) 2978, 3364 (Ar CH), (-OH stretch) 3494,

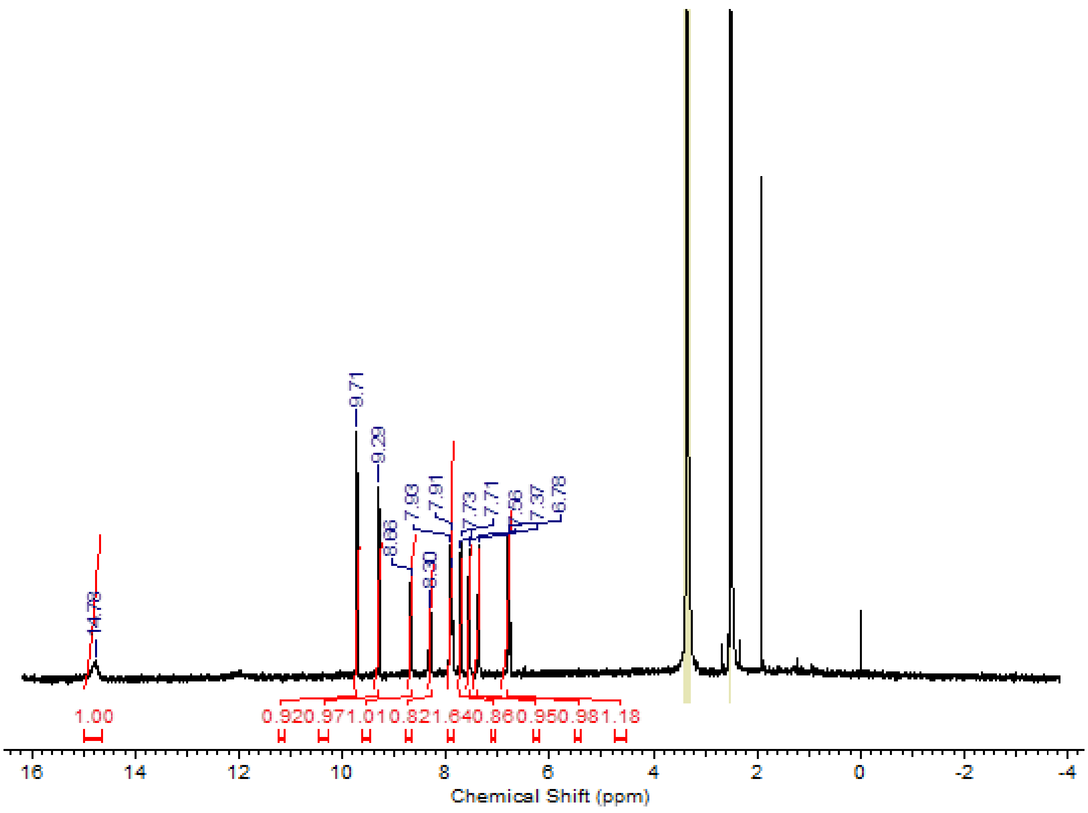

Figure S5: ^{1}H NMR spectrum of receptor **R2**

^{1}H NMR (DMSO-d_6, 400 MHz, ppm): δ 14.78 (s,OH), 9.71 (s, Ar-H), 9.29 (s, Ar-H), 8.66 (s,CH=N), 8.30(d, Ar-H), 7.92 (d, Ar-H), 7.72 (d, Ar-H), 7.71 (s, Ar-H), 7.56 (s,Ar-H), 7.37(s,Ar-H), 6.78 (s, Ar-H)

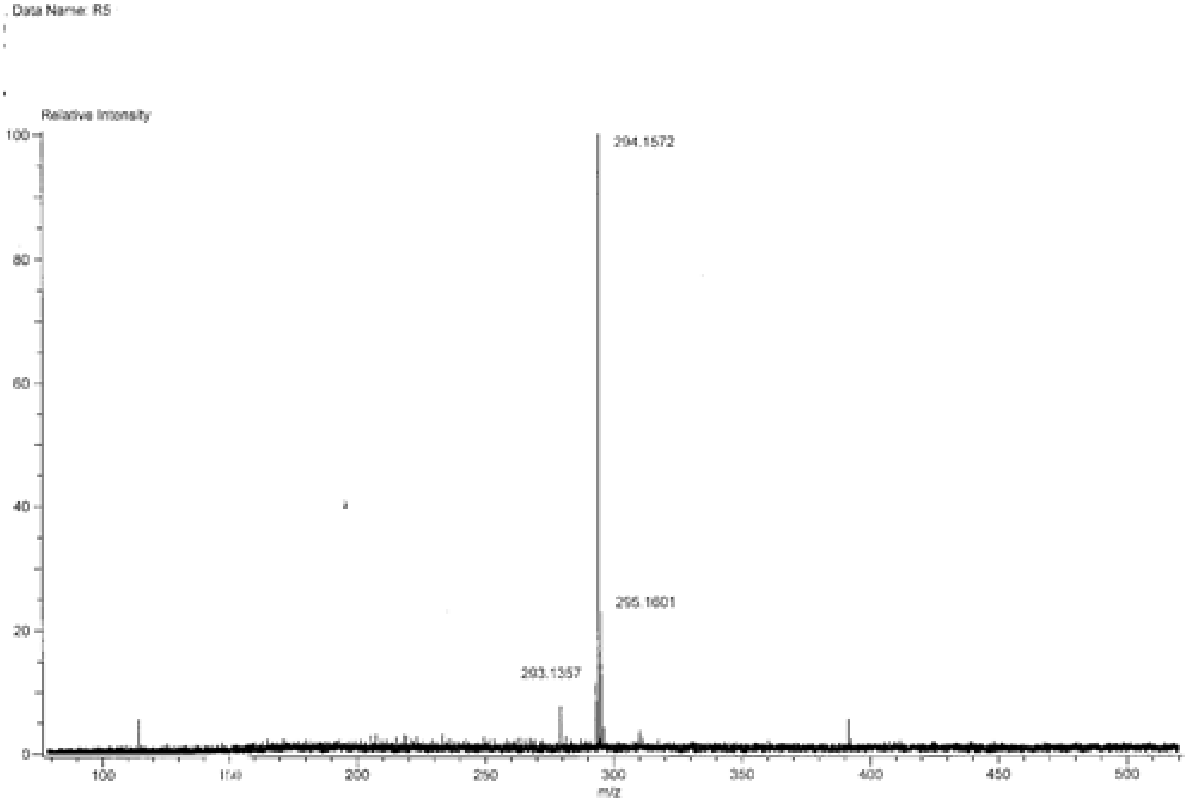

Figure S6: Mass spectrum of receptor **R2**

Calculated: 293.08

Obtained: (M+ H $^{+}$) 294.15

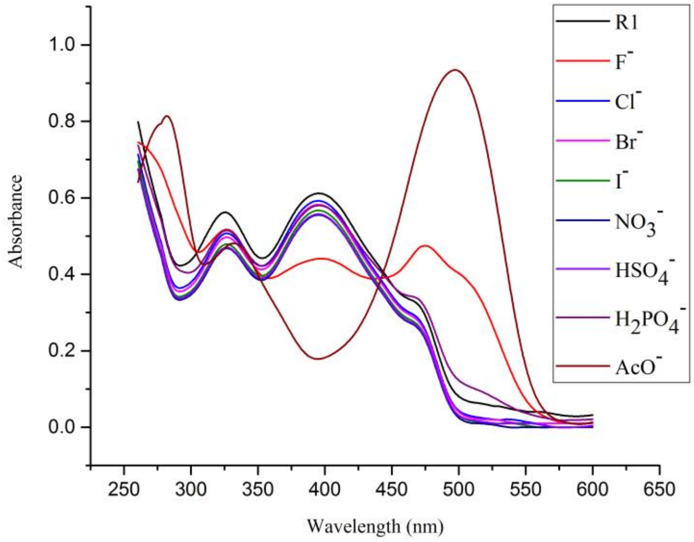

Figure S7: UV–vis absorption spectra of **R1** (4.5×10^{-5} M in DMSO) upon addition of 1 equiv of various anions as TBA salts

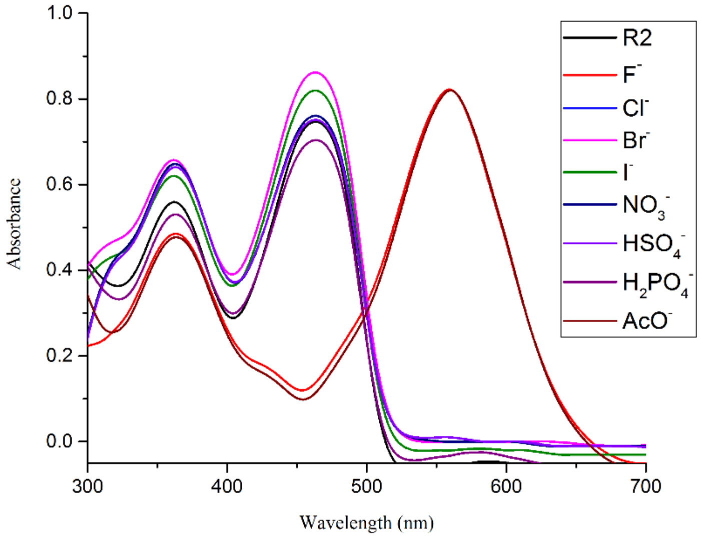

Figure S8: UV–vis absorption spectra of **R2** (4.5×10^{-5} M in DMSO) upon addition of 1 equiv of various anions as TBA salts

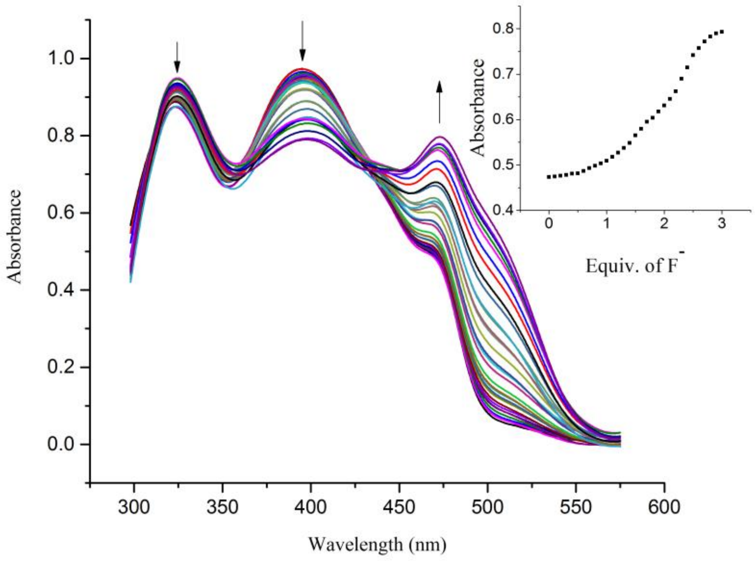

Figure S9: UV–vis titration spectra of receptor **R1** (4.5×10^{-5} M in DMSO/H_2O 9:1 (v/v)) with the addition of 0.1 equiv of NaF (1×10^{-2} M in distilled water). Inset plot represents the binding isotherm at 473 nm.

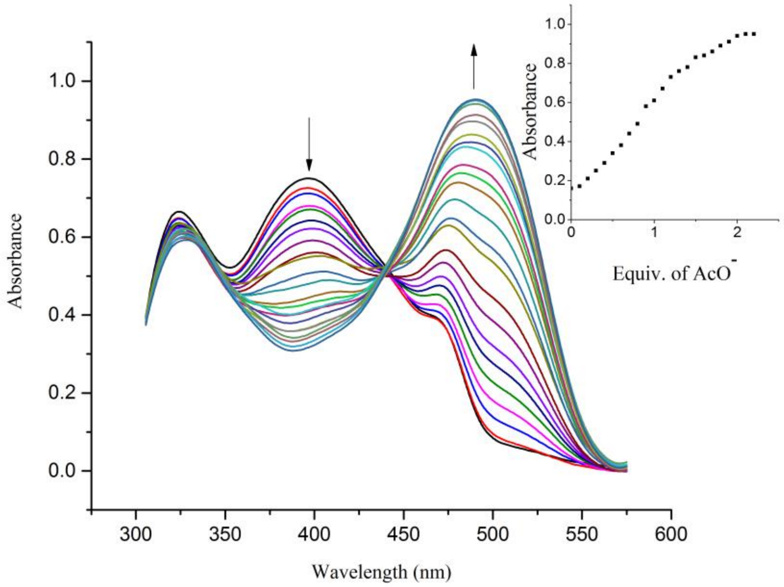

Figure S10: UV–vis titration spectra of receptor **R1** (4.5×10^{-5} M in DMSO/H_2O 9:1 (v/v)) with the addition of 0.1 equiv of NaAcO (1×10^{-2} M in distilled water). Inset plot represents the binding isotherm at 489 nm

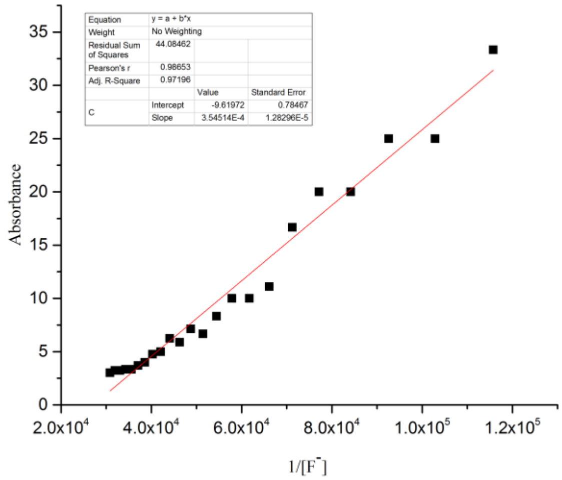

Figure S11: B–H plot of receptor **R1**-F^{-} (as NaF) complex at a selected wavelength of 473 nm

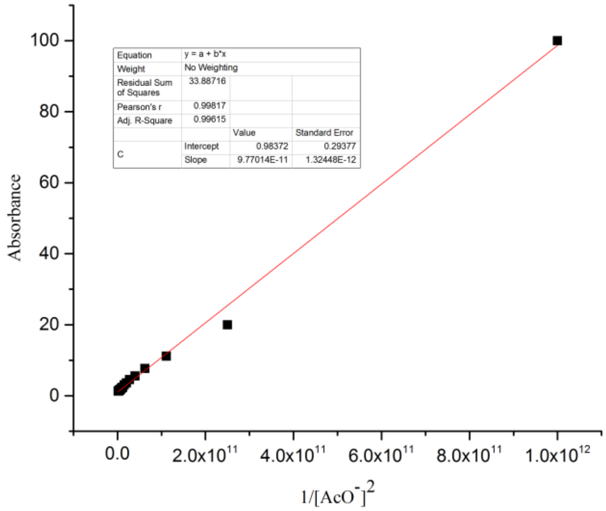

Figure S12: B–H plot of receptor **R1**-AcO^{-} (NaAcO) complex at a selected wavelength of 489 nm

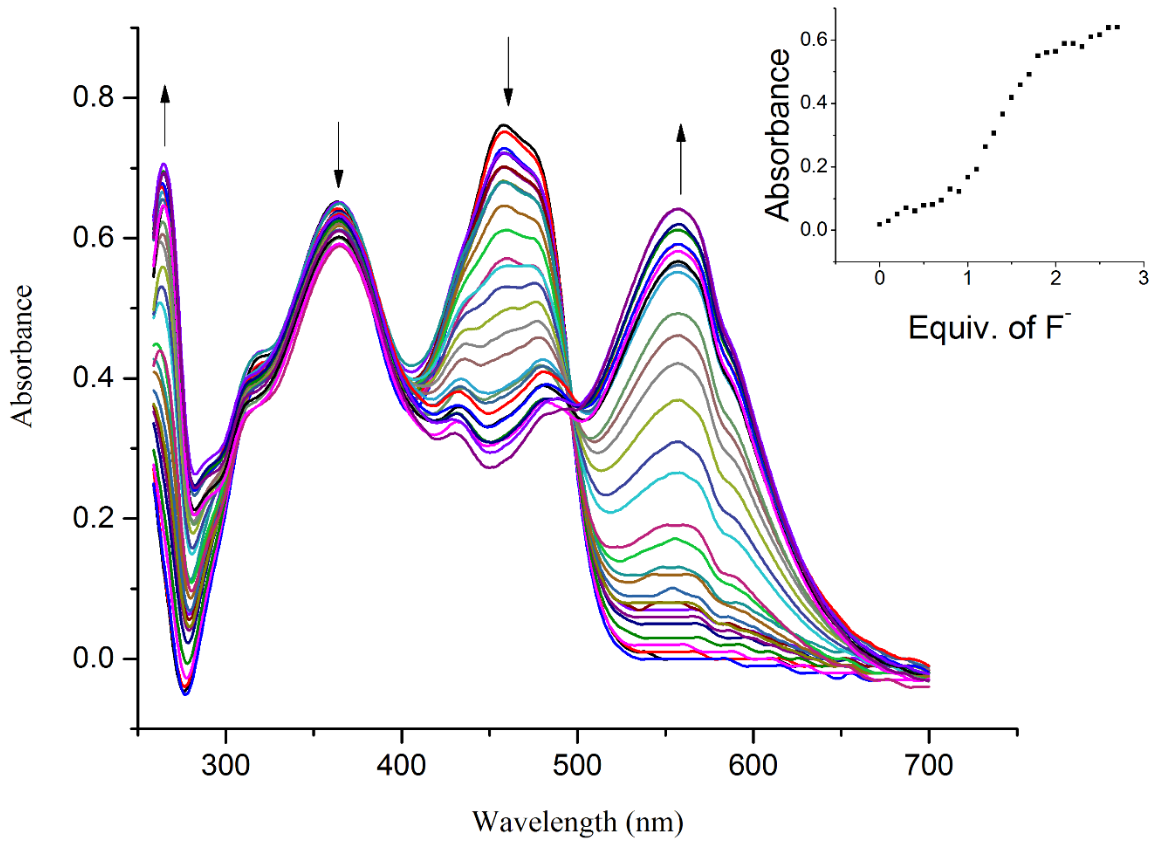

Figure S13: UV–vis titration spectra of receptor **R2** (4.5×10^{-5} M in DMSO/H_2O 9:1 (v/v)) with the addition of 0.1 equiv of NaF (1×10^{-2} M in distilled water). Inset plot represents the binding isotherm at 556 nm

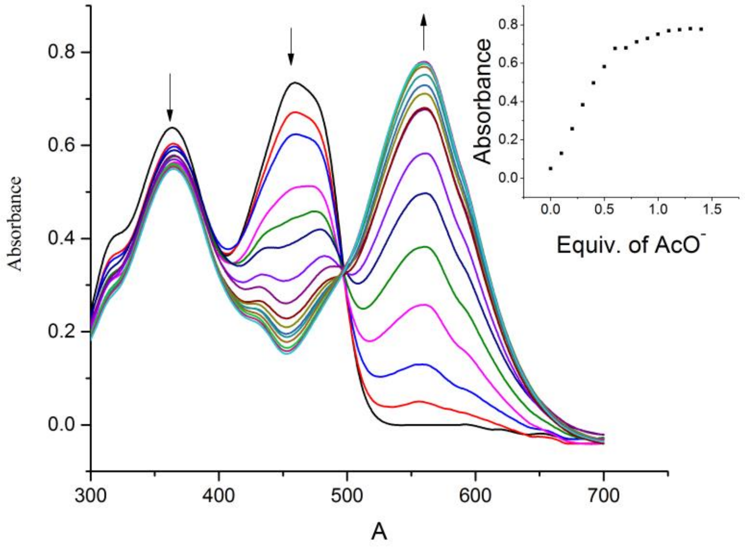

Figure S14: UV–vis titration spectra of receptor **R2** (4.5×10^{-5} M in DMSO/H_2O 9:1 (v/v)) with the addition of 0.1 equiv of NaAcO (1×10^{-2} M in distilled water). Inset plot represents the binding isotherm at 559 nm

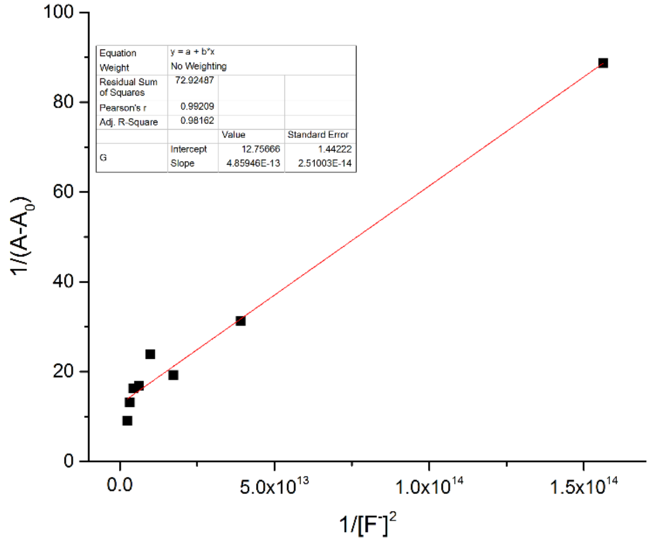

Figure S15: B–H plot of receptor **R2**-F^- (NaF) complex at a selected wavelength of 556 nm

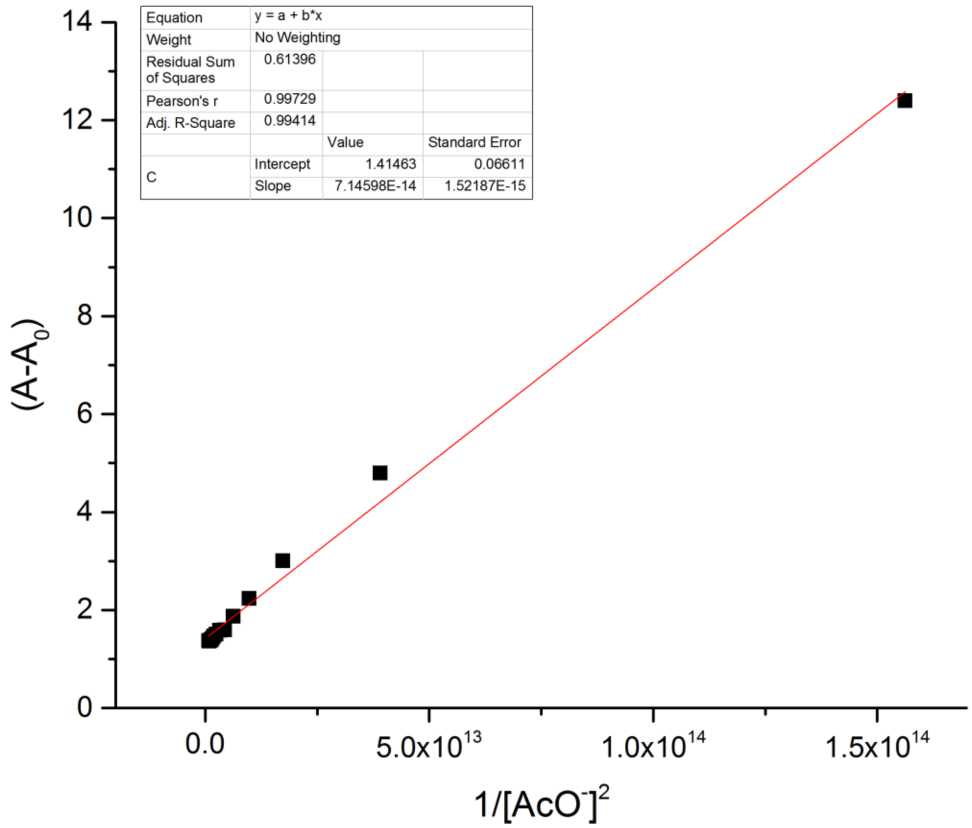

Figure S16: B–H plot of receptor **R2**-AcO^{-} (NaAcO) complex at a selected wavelength of 559 nm

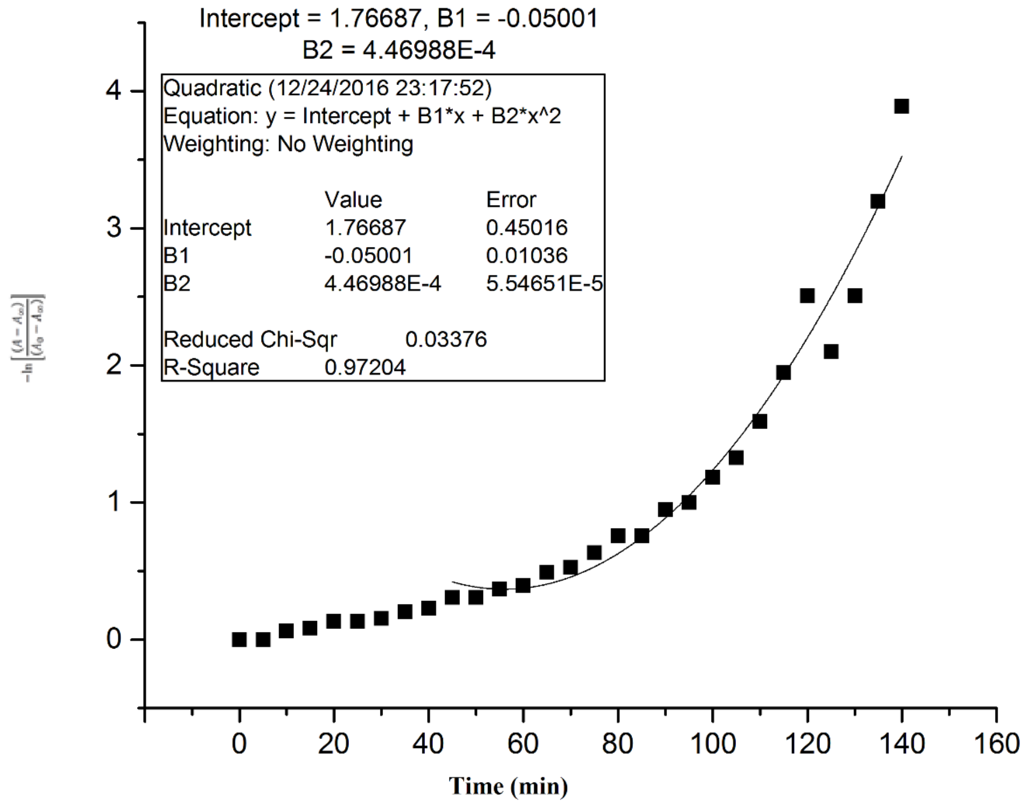

Figure S17: Time dependency plot of first order rate equation to determine the rate constant from UV–vis spectral change of **R1** in the presence of F^{-} ion at 492 nm

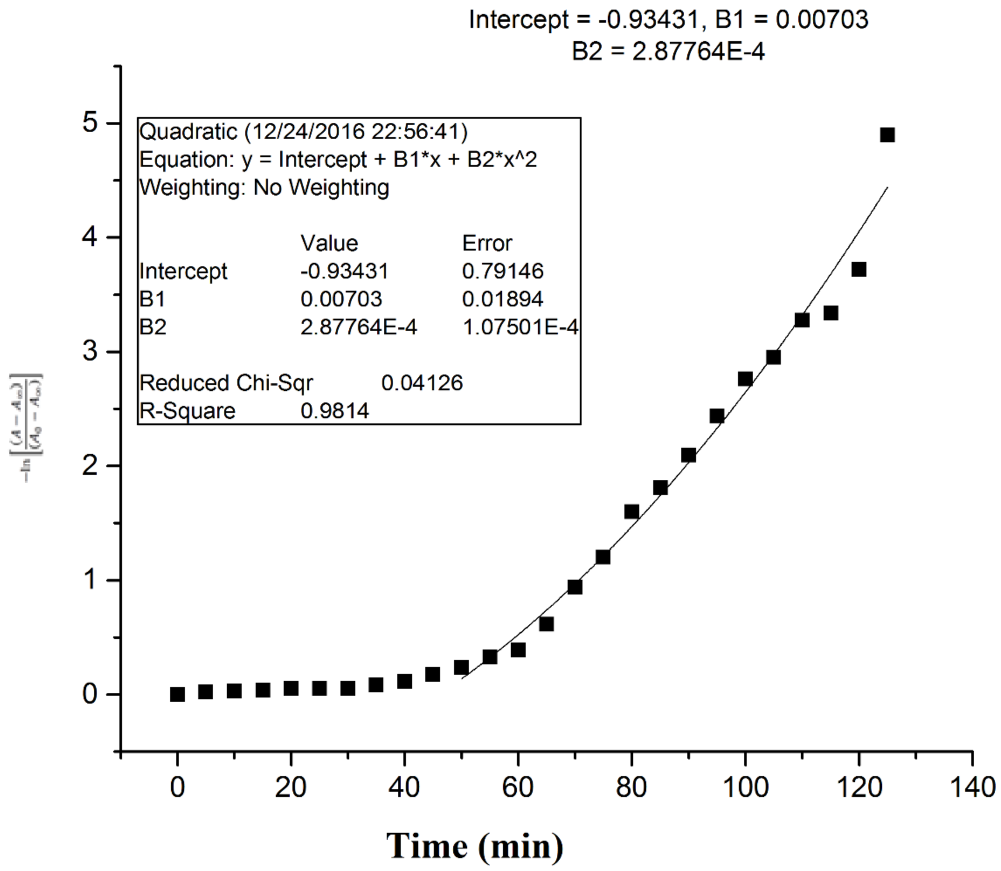

Figure S18: Time dependency plot of first order rate equation to determine the rate constant from UV–vis spectral change of **R2** in the presence of F^{-} ion at 560 nm

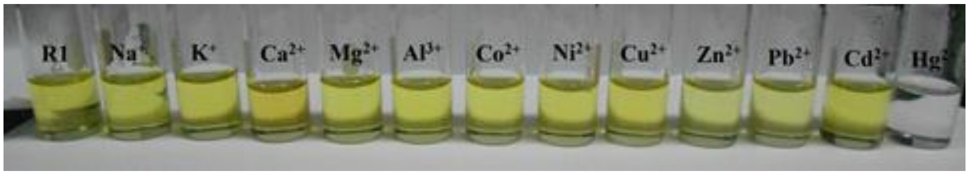

Figure S19: Color change of receptor **R1** (4.5×10^{-5} M in DMSO) upon the addition of 1 equiv of a variety of cations (10^{-3} M in distilled water)

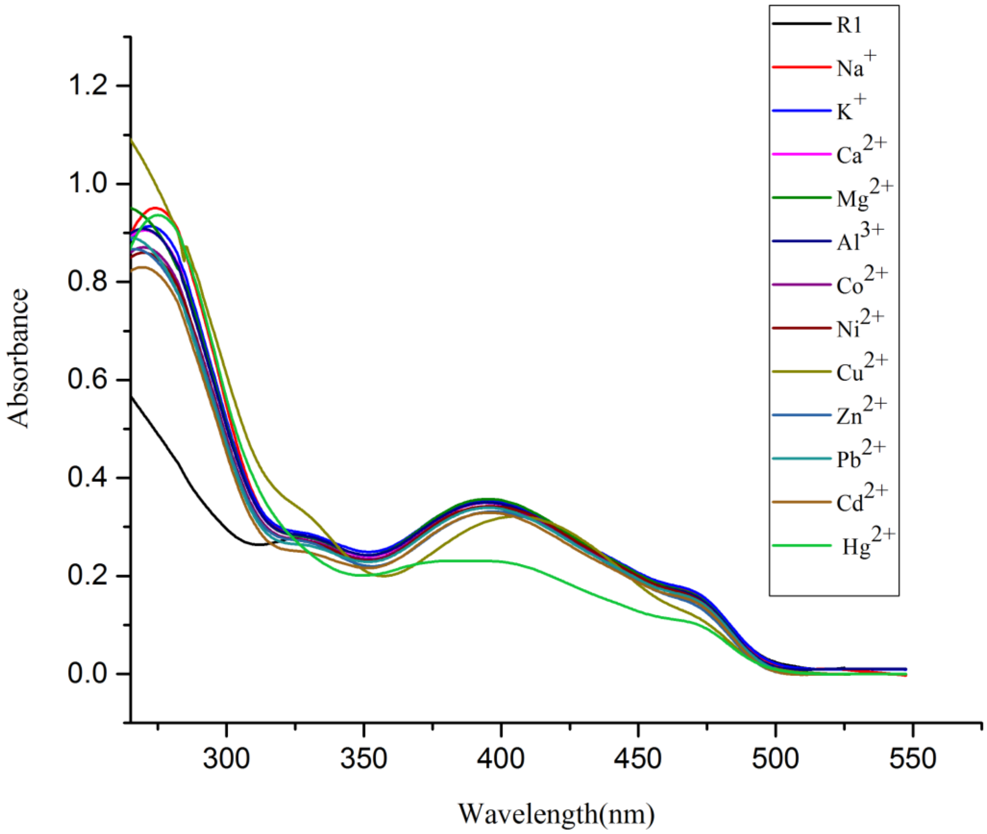

Figure S20: UV–vis absorption spectra of **R1** (4.5×10^{-5} M in DMSO) in the presence of different cations (1×10^{-3} M in distilled water)

Table S1: Mulliken charge distribution of **R1, R1**+F^{-}, **R1**+AcO^{-} and **R2**, **R2**+F^{-}, **R2**+AcO^{-}

**Receptor 1**				**Receptor 1 + F^-**				**Receptor 1 + AcO^-**		
**No**	**Atom**	**Charge**		**No**	**Atom**	**Charge**		**No**	**Atom**	**Charge**
1	C	0.586062		1	C	-0.08016		1	C	-0.04695
2	C	-0.46591		2	C	-0.3762		2	C	-0.55086
3	N	-0.00382		3	N	-0.0318		3	N	-0.00661
4	C	0.882446		4	C	0.957446		4	C	0.944994
5	C	-0.10687		5	C	0.002467		5	C	0.110502
6	C	0.169448		6	C	0.138339		6	C	0.001159

7	N	0.145243		7	N	0.092191		7	N	0.114041
8	C	0.002938		8	C	0.109565		8	C	0.094488
9	C	0.782404		9	C	0.460322		9	C	0.290122
10	C	-0.01642		10	C	0.258786		10	C	0.367299
11	C	0.283046		11	C	0.325158		11	C	0.380053
12	C	0.077189		12	C	-0.09579		12	C	-0.12567
13	C	-0.5382		13	C	-0.35094		13	C	-0.20553
14	C	-1.29525		14	C	-0.73343		14	C	-0.7703
15	C	-0.36336		15	C	-0.4643		15	C	-0.46756
16	C	0.018342		16	C	-0.17499		16	C	-0.24191
17	C	-0.46703		17	C	-0.38956		17	C	-0.38981
18	C	-0.18171		18	C	-0.35688		18	C	-0.37646
19	O	-0.18186		19	O	-0.45334		19	O	-0.56776
20	C	-1.33956		20	C	-1.34836		20	C	-1.29279
21	N	-0.27929		21	N	-0.29718		21	N	-0.30091
22	H	0.241903		22	H	0.26785		22	H	0.333495
23	H	0.272001		23	H	0.250134		23	H	0.248221
24	H	0.218337		24	H	0.216735		24	H	0.214093
25	H	0.120017		25	H	0.117467		25	H	0.208498
26	H	0.184085		26	H	0.173854		26	H	0.171792
27	H	0.219509		27	H	0.192138		27	H	0.182683
28	H	0.171757		28	H	0.174451		28	H	0.16713
29	H	0.199305		29	H	0.1896		29	H	0.190969
30	H	0.190831		30	H	0.179371		30	H	0.178957
31	H	0.176882		31	H	0.159121		31	H	0.158342
32	H	0.297526		32	H	0.247477		32	H	0.576031
				33	F	-0.35956		33	C	0.267252
								34	O	-0.3634
								35	O	-0.37542
								36	C	-0.64543

								37	H	0.181752
								38	H	0.160974
								39	H	0.184525
**Receptor 2**				**Receptor 2 + F^-**				**Receptor 2 + AcO^-**		
No	Atom	**Charge**		**No**	**Atom**	**Charge**		**No**	**Atom**	**Charge**
1	C	-0.61387		1	C	-0.5317		1	C	-0.34884
2	C	-0.3157		2	C	-0.35419		2	C	-0.41591
3	C	-0.14527		3	C	-0.14634		3	C	-0.29949
4	C	0.158578		4	C	0.05685		4	C	0.141298
5	C	0.442141		5	C	0.507848		5	C	0.465076
6	C	-0.83494		6	C	-0.71588		6	C	-0.6118
7	C	-0.13285		7	C	-0.11485		7	C	-0.1322
8	C	-0.38149		8	C	-0.23825		8	C	-0.38355
9	C	0.089474		9	C	-0.34317		9	C	-0.19177
10	C	0.431653		10	C	0.562489		10	C	0.522469
11	C	-0.45084		11	C	-0.18038		11	C	-0.01441
12	N	-0.0499		12	N	-0.11777		12	N	-0.02453
13	O	-0.33913		13	O	-0.52996		13	O	-0.47443
14	C	0.381355		14	C	0.175413		14	C	-0.49006
15	C	-0.20391		15	C	-0.12475		15	C	0.113302
16	C	0.408323		16	C	-0.08372		16	C	0.06431
17	C	-1.12911		17	C	-0.52001		17	C	-0.49736
18	C	0.288779		18	C	0.125396		18	C	0.146925
19	N	-0.06037		19	N	-0.0803		19	N	-0.11914
20	N	-0.12947		20	N	-0.19139		20	N	-0.18921
21	O	-0.05318		21	O	-0.08202		21	O	-0.07702
22	O	-0.07045		22	O	-0.10532		22	O	-0.10976
23	H	0.215043		23	H	0.201		23	H	0.198831
24	H	0.187772		24	H	0.178177		24	H	0.177281
25	H	0.170712		25	H	0.160223		25	H	0.162323

26	H	0.047996		26	H	0.044009		26	H	0.073293
27	H	0.189		27	H	0.173861		27	H	0.174232
28	H	0.203097		28	H	0.186879		28	H	0.176894
29	H	0.338196		29	H	0.241124		29	H	0.15656
30	H	0.569339		30	H	0.44129		30	H	0.593884
31	H	0.210186		31	H	0.257893		31	H	0.238086
32	H	0.292435		32	H	0.266538		32	H	0.264337
33	H	0.286395		33	H	0.274457		33	H	0.273767
				34	F	-0.39347		34	C	0.026539
								35	O	-0.35414
								36	O	-0.25264
								37	C	-0.51303
								38	H	0.184756
								39	H	0.178162
								40	H	0.166971

Calculation of binding constants:

Binding constant has been calculated using equation[1] (A1).

$$\mathbf{1/(A-A_o) = 1/(A_{max} - A_o) + 1/K\,[F^-]^n\,(A_{max} - A_o)} \quad \text{------------------} \quad \mathbf{(A1)}$$

Where, A_0, A, A_{max} are the absorption considered in the absence of F^-, at an intermediate, and at a concentration of saturation. K is binding constant, $[F^-]$ is concentration of F^- ion and n is the stoichiometric ratio.

Calculation of detection limit:

The limit of detection was found using this equation.[2]

$DL = C_L \times C_T$

C_L = Conc. of receptor; C_T = Conc. of Titrant at which change observed.

References:

1. H. Benesi and H. Hildebrand, J. Am. Chem. Soc., 1948, 71, 2703–2707.
2. V. Bhalla, A. Gupta, M. Kumar., Chem. Commun., 2012, 48, 11862.
